# Assessing Causality in the Association between Child Adiposity and Physical Activity Levels: A Mendelian Randomization Analysis

**DOI:** 10.1371/journal.pmed.1001618

**Published:** 2014-03-18

**Authors:** Rebecca C. Richmond, George Davey Smith, Andy R. Ness, Marcel den Hoed, George McMahon, Nicholas J. Timpson

**Affiliations:** 1 MRC Integrative Epidemiology Unit, School of Social and Community Medicine, University of Bristol, Bristol, United Kingdom; 2 Department of Oral and Dental Science, University of Bristol, Bristol, United Kingdom; 3 Molecular Epidemiology and Science for Life Laboratory, Department of Medical Sciences, Uppsala University, Uppsala, Sweden; Boston Children's Hospital, United States of America

## Abstract

**Background:**

Cross-sectional studies have shown that objectively measured physical activity is associated with childhood adiposity, and a strong inverse dose–response association with body mass index (BMI) has been found. However, few studies have explored the extent to which this association reflects reverse causation. We aimed to determine whether childhood adiposity causally influences levels of physical activity using genetic variants reliably associated with adiposity to estimate causal effects.

**Methods and Findings:**

The Avon Longitudinal Study of Parents and Children collected data on objectively assessed activity levels of 4,296 children at age 11 y with recorded BMI and genotypic data. We used 32 established genetic correlates of BMI combined in a weighted allelic score as an instrumental variable for adiposity to estimate the causal effect of adiposity on activity.

In observational analysis, a 3.3 kg/m^2^ (one standard deviation) higher BMI was associated with 22.3 (95% CI, 17.0, 27.6) movement counts/min less total physical activity (*p* = 1.6×10^−16^), 2.6 (2.1, 3.1) min/d less moderate-to-vigorous-intensity activity (*p* = 3.7×10^−29^), and 3.5 (1.5, 5.5) min/d more sedentary time (*p* = 5.0×10^−4^). In Mendelian randomization analyses, the same difference in BMI was associated with 32.4 (0.9, 63.9) movement counts/min less total physical activity (*p* = 0.04) (∼5.3% of the mean counts/minute), 2.8 (0.1, 5.5) min/d less moderate-to-vigorous-intensity activity (*p* = 0.04), and 13.2 (1.3, 25.2) min/d more sedentary time (*p* = 0.03). There was no strong evidence for a difference between variable estimates from observational estimates. Similar results were obtained using fat mass index. Low power and poor instrumentation of activity limited causal analysis of the influence of physical activity on BMI.

**Conclusions:**

Our results suggest that increased adiposity causes a reduction in physical activity in children and support research into the targeting of BMI in efforts to increase childhood activity levels. Importantly, this does not exclude lower physical activity also leading to increased adiposity, i.e., bidirectional causation.

*Please see later in the article for the Editors' Summary*

## Abstract 2

Here, Timpson and colleagues performed a Mendelian Randomization analysis to determine whether childhood adiposity causally influences levels of physical activity. The results suggest that increased adiposity causes a reduction in physical activity in children; however, this study does not exclude lower physical activity also leading to increasing adiposity.

*Please see later in the article for the Editors' Summary*

## Editors' Summary

**Background:**

The World Health Organization estimates that globally at least 42 million children under the age of five are obese. The World Health Organization recommends that all children undertake at least one hour of physical activity daily, on the basis that increased physical activity will reduce or prevent excessive weight gain in children and adolescents. In practice, while numerous studies have shown that body mass index (BMI) shows a strong inverse correlation with physical activity (i.e., active children are thinner than sedentary ones), exercise programs specifically targeted at obese children have had only very limited success in reducing weight. The reasons for this are not clear, although environmental factors such as watching television and lack of exercise facilities are traditionally blamed.

**Why Was This Study Done?:**

One of the reasons why obese children do not lose weight through exercise might be that being fat in itself leads to a decrease in physical activity. This is termed reverse causation, i.e., obesity causes sedentary behavior, rather than the other way around. The potential influence of environmental factors (e.g., lack of opportunity to exercise) makes it difficult to prove this argument. Recent research has demonstrated that specific genotypes are related to obesity in children. Specific variations within the DNA of individual genes (single nucleotide polymorphisms, or SNPs) are more common in obese individuals and predispose to greater adiposity across the weight distribution. While adiposity itself can be influenced by many environmental factors that complicate the interpretation of observed associations, at the population level, genetic variation is not related to the same factors, and over the life course cannot be changed. Investigations that exploit these properties of genetic associations to inform the interpretation of observed associations are termed Mendelian randomization studies. This research technique is used to reduce the influence of confounding environmental factors on an observed clinical condition. The authors of this study use Mendelian randomization to determine whether a genetic tendency towards high BMI and fat mass is correlated with reduced levels of physical activity in a large cohort of children.

**What Did the Researchers Do and Find?:**

The researchers looked at a cohort of children from a large long-term health research project (the Avon Longitudinal Study of Parents and Children). BMI and total body fat were recorded. Total daily activity was measured via a small movement-counting device. In addition, the participants underwent genotyping to detect the presence of several SNPs known to be linked to obesity. For each child a total BMI allelic score was determined based on the number of obesity-related genetic variants carried by that individual. The association between obesity and reduced physical activity was then studied in two ways. Direct correlation between actual BMI and physical activity was measured (observational data). Separately, the link between BMI allelic score and physical activity was also determined (Mendelian randomization or instrumental variable analysis). The observational data showed that boys were more active than girls and had lower BMI. Across both sexes, a higher-than-average BMI was associated with lower daily activity. In genetic analyses, allelic score had a positive correlation with BMI, with one particular SNP being most strongly linked to high BMI and total fat mass. A high allelic score for BMI was also correlated with lower levels of daily physical activity. The authors conclude that children who are obese and have an inherent predisposition to high BMI also have a propensity to reduced levels of physical activity, which may compound their weight gain.

**What Do These Findings Mean?:**

This study provides evidence that being fat is in itself a risk factor for low activity levels, separately from external environmental influences. This may be an example of “reverse causation,” i.e., high BMI causes a reduction in physical activity. Alternatively, there may be a bidirectional causality, so that those with a genetic predisposition to high fat mass exercise less, leading to higher BMI, and so on, in a vicious circle. A significant limitation of the study is that validated allelic scores for physical activity are not available. Thus, it is not possible to determine whether individuals with a high allelic score for BMI also have a propensity to exercise less, or whether it is simply the circumstance of being overweight that discourages activity. This study does suggest that trying to persuade obese children to lose weight by exercising more is likely to be ineffective unless additional strategies to reduce BMI, such as strict diet control, are also implemented.

**Additional Information:**

Please access these websites via the online version of this summary at http://dx.doi.org/10.1371/journal.pmed.1001618.

- The US Centers for Disease Control and Prevention provides obesity-related statistics, details of prevention programs, and an overview on public health strategy in the United States
- A more worldwide view is given by the World Health Organization
- The UK National Health Service website gives information on physical activity guidelines for different age groups
- The International Obesity Task Force is a network of organizations that seeks to alert the world to the growing health crisis threatened by soaring levels of obesity
- MedlinePlus—which brings together authoritative information from the US National Library of Medicine, National Institutes of Health, and other government agencies and health-related organizations—has a page on obesity
- Additional information on the Avon Longitudinal Study of Parents and Children is available
- The *British Medical Journal* has an article that describes Mendelian randomization

## Introduction

Cross-sectional studies have shown that objectively measured physical activity is associated with childhood adiposity [1]–[6], and a strong inverse dose–response association with body mass index (BMI) has been found [1]. However, confounding or reverse causation (where adiposity influences inactivity, rather than vice versa) may explain part of the association [7],[8]. Indeed, there may be a bidirectional relationship between adiposity and physical activity, and this would imply that only a small change in adiposity or physical activity may be required to initiate a cycle of weight gain and increased inactivity [9].

There are few randomized trials examining the effectiveness of physical activity interventions for weight loss [10]. Those that exist report smaller, if any, effects on BMI [11]–[14] than predicted by observational associations. However, the efficacy of BMI as a measure of adiposity is subject to debate, and some improvements in other measures of fatness such as skinfold thickness have been demonstrated in school-based physical activity interventions, without an accompanying reduction in BMI [14]. Nevertheless, the small effect seen in these trials suggests that reverse causation may in part have generated the association between physical activity and adiposity observed in cross-sectional studies. These findings, together with the quality of the trials—which has been limited by short trial duration, lack of assessment of trial adherence, or a limited difference in activity achieved between intervention and control groups [12]—call for further investigation and the use of genetic instruments as a better surrogate for adiposity.

To address the issue of reverse causation, prospective studies have measured activity and adiposity at multiple time points in children [7],[15]–[22], although few studies have investigated bidirectional associations between activity and fatness in childhood and adolescence. Of those that have, one showed a lack of longitudinal association between physical activity and body composition [21], while three showed that whereas physical activity could not predict fatness, fatness was predictive of future physical inactivity [7],[20],[22]. Sample size and poorly assessed activity have limited the ability to infer the causal direction of effects, even where longitudinal data are available.

Mendelian randomization (MR) can be used to assess whether adiposity causally affects activity levels [23]. MR is an approach that applies instrumental variable methods, using genetic variants as a proxy for environmentally modifiable exposures. This technique, which is analogous to a randomized trial where randomization to genotype takes place at conception, is not susceptible to reverse causation or confounding and so may be used to reassess observational associations and strengthen causal inference [23]–[26].

**Figure 1:**
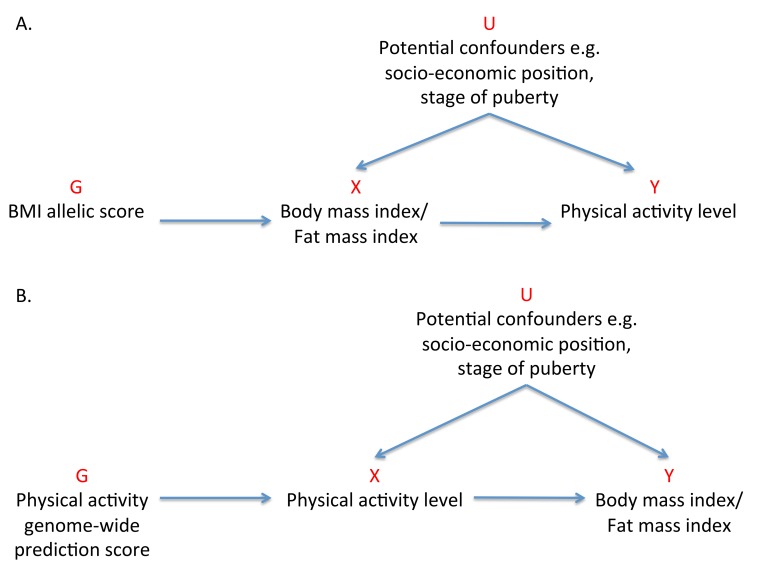
Addressing the causal directions of effect in the association between adiposity and physical activity with the use of allelic scores and Mendelian randomization analysis. (A) MR analysis to investigate the causal effect of adiposity on levels of physical activity with the use of a weighted allelic score as a genetic instrument. (B) Reciprocal MR analysis to investigate the causal effects of physical activity levels on adiposity using a genome-wide prediction score as a genetic instrument. G, genetic instrument; U, unobserved confounders; X, exposure; Y, outcome.

Previous MR studies investigating the effect of adiposity on various outcomes have used one or a few of the common genetic locus variants with the largest effect sizes to serve as instruments [27]–[32]. In this study, we aimed to use 32 independent genetic correlates of BMI, confirmed in a large-scale meta-analysis of genome-wide association studies (GWASs) [33], to elucidate the causality and magnitude of the effect of adiposity on activity levels in children (Figure 1A).

## Methods

### Study Sample

The Avon Longitudinal Study of Parents and Children (ALSPAC) is a prospective birth cohort that enrolled over 13,000 pregnant women in the former County of Avon, UK, with an expected delivery date between April 1991 and December 1992 [34],[35]. Detailed information has been collected on these women and their offspring using self-administered questionnaires, research clinic examinations, data extraction from medical notes, and linkage to routine information systems. The study website contains details of all available data through a fully searchable data dictionary (http://www.bristol.ac.uk/alspac/researchers/data-access/data-dictionary/). Ethical approval was obtained from the ALSPAC Law and Ethics Committee and local research ethics committees.

### Exposure Variables

Body composition was measured at a clinic where the children's average age was 11.7 y [1]. BMI, the primary exposure variable, was calculated as weight (in kilograms) divided by height (in meters) squared. BMI as a measure of adiposity has well-recognised limitations [36],[37], and of particular concern for this analysis is that BMI does not distinguish between fat and lean mass, since lean mass correlates positively with levels of activity [2]. Therefore, phenotypic refinement was employed through the use of total body fat, assessed using a Lunar Prodigy dual energy X-ray absorptiometry scanner [1]. The fat mass index (FMI) was subsequently calculated as fat mass (in kilograms) divided by height (in meters) squared.

### Outcome Variables

All children who attended the age 11-y clinic were asked to wear an MTI Actigraph AM7164 2.2 accelerometer for 7 d [38]. Only data from children who wore the Actigraph for at least 10 h/d for 3 d were included in this analysis. Movement counts were detected as a combined function of the frequency and intensity of movements. Activity was expressed as the total daily volume of physical activity averaged over the period of valid recording (counts/minute), and as time spent on moderate-to-vigorous-intensity physical activity (>3,600 counts/min) [39] and sedentary time (<199 counts/min) in minutes/day [40].

### Genotyping

9,912 ALSPAC children were genotyped using the Illumina HumanHap550 quad genome-wide single nucleotide polymorphism (SNP) genotyping platform by the Wellcome Trust Sanger Institute (Cambridge, UK) and the Laboratory Corporation of America (Burlington, North Carolina, US). Individuals with incorrect sex assignments, extreme heterozygosity (<0.320 and >0.345 for Wellcome Trust Sanger Institute data and <0.310 and >0.330 for Laboratory Corporation of America data), disproportionate levels of individual missingness (>3%), evidence of cryptic relatedness (>10% identity by descent), or non-European ancestry were excluded. The resulting dataset consisted of 8,365 individuals. Of 609,203 SNPs, those with a minor allele frequency of <1%, with a call rate of <95%, or not in Hardy–Weinberg equilibrium (*p*<5×10^−7^) were removed, leaving 500,527 SNPs that passed quality control. Established BMI variants that had not been genotyped directly were imputed with MACH 1.0.16 Markov Chain Haplotyping software [41],[42] using CEPH individuals from HapMap phase 2 (release 22) as a reference set.

From these genome-wide data, a weighted allelic score was created using 32 independent variants shown to be robustly associated with BMI in a large-scale GWAS meta-analysis [33] (Table S1). The dose of the effect allele at each locus was weighted by the effect size of the variant in this independent meta-analysis [33], and these doses were summed to reflect the average number of BMI-increasing alleles carried by an individual. This weighted allelic score was created to act as an instrumental variable in MR analysis, and explained a greater proportion of variance in BMI than single SNPs [43]. The allelic score was also used as an instrument for FMI.

### Statistical Methods

Means and standard deviations (SD) were calculated for continuous variables to describe baseline characteristics. The distribution of moderate-to-vigorous activity was skewed and was therefore log-transformed to achieve normality. All adiposity and activity values were converted to sex-specific SD (*z*) scores.

Observational associations between adiposity and activity measures were assessed using linear regression adjusted for age. Additional analyses were adjusted for potentially confounding factors that have been found to be independently associated with obesity [44], including maternal pre-pregnancy BMI, estimated gestational age at birth, infant birth weight, maternal education level, parental social class, maternal smoking during pregnancy, child's stage of puberty at age 11 y, total daily dietary intake, and intake of main food groups.

For investigating associations between the allelic score and standardised phenotypes, continuous effects were estimated using linear regression with adjustment for age. An additive genetic model was assumed since there was no evidence for interaction effects among the SNPs combined in the allelic score [33]. MR analysis may generally forego the need for inclusion of other covariates, which are anticipated to be randomly distributed with respect to genotype [23]. Despite this, we examined associations between the confounding factors and genotypes to check the core instrumental variable assumption that the instrument (genotype) is independent of factors that potentially confound the observational association [25],[26].

For MR analyses, we performed two-stage least squares using the weighted allelic score as an instrument for adiposity and implementing the “ivreg2” function in Stata. *F*-statistics from the first-stage regression between genotype and adiposity were examined to check the instrumental variable assumption that the instrument is sufficiently associated with the exposure, in order to reduce the possibility of weak instrument bias [45]. The Durbin-Wu-Hausman (DWH) test for endogeneity [46] was used to compare effect estimates from the second stage of the instrumental variable analysis and observational analysis. Stata 12 (StataCorp) was used for all analyses.

### Sensitivity Analyses

#### Multiple independent instruments

The existence of pleiotropy, where a genetic instrument has an effect on an outcome (activity) independent of its effect on the exposure (adiposity), would have implications for assumptions made in MR analyses [47]. Similar instrumental variable estimates acquired using independent instruments would provide suggestive evidence against an influence of pleiotropic effects, as it is unlikely that they have shared pleiotropy [43],[48]. The two independent genetic instruments generated were rs1558902 in *FTO*, the individual SNP with the largest effect size in the meta-analysis of GWASs for BMI [33], and a weighted allelic score constructed from the remaining 31 BMI-associated SNPs.

#### Genome-wide prediction for physical activity

An exploratory MR analysis investigating the association between adiposity and activity levels may provide evidence for causality in this direction. However, it does not exclude the possibility that physical activity has a causal effect on adiposity levels. A genetic instrument for activity is required to test the relationship in a bidirectional manner (Figure 1B) [31],[32]. No meta-analysis of GWASs has so far been reported for physical activity, and no genetic variants have been robustly associated with activity to date [49],[50]. Genome-wide prediction scores, which examine the aggregated contribution of genome-wide variation in a trait, have the potential to recover some of the information lost by dismissing false-negative results in GWASs [51]–[55] and may be used as instruments in MR analysis.

Before the generation of a genetic instrument for physical activity, the heritability of activity in ALSPAC was assessed to consider the plausibility of a genetic contribution to activity. GCTA (Genome-wide Complex Trait Analysis) (version 1.04) [56] was used to estimate the total amount of variance captured by all 500,527 SNPs in the genotypic data for the activity measures. The approach first involves the estimation of a genetic relationship matrix for individuals based on autosomal genotype information, with a further cryptic relatedness cutoff of 2.5% applied to reduce the potential for biased estimates. The variance of each activity trait attributable to all SNPs was estimated using restricted maximum likelihood. Given evidence of a heritable contribution to observed variance in activity measures (Table S2), genome-wide prediction scores were generated for total physical activity, moderate-to-vigorous activity, and sedentary time. Individuals in the complete sample were randomized into two subgroups. Using activity and genotypic data from the first subgroup (*n* = 2,148), genetic variants yielding a *p*-value≤0.1 in a GWAS for each activity variable were extracted, and prediction scores were constructed using profile scoring and the “–score” command within PLINK (version 1.07) [57]. The prediction score is a sum across SNPs of the number of reference alleles multiplied by the weight for that SNP, which is its effect size in the GWAS with activity.

We used split sample analysis, where the physical activity prediction scores from the first subgroup (composed of one half of the sample) were applied to individuals in the second independent subgroup (composed of the other half of the sample) and used in two-stage least squares instrumental variable analysis to assess a causal effect of activity on adiposity. This method was repeated with prediction scores generated from data in the second subgroup and applied to instrumental variable analysis in the first [58]. The results of these two instrumental variable analyses were meta-analysed using the inverse variance-weighted method with a fixed-effects model. A test for heterogeneity [59] was performed to investigate similarity between instrumented effects in the two independent subgroups.

We performed all of the above analyses stratified by sex because a sex interaction for the associations between adiposity and activity levels has been shown previously [1] (Tables S11, S12, S13).

## Results

**Figure 2:**
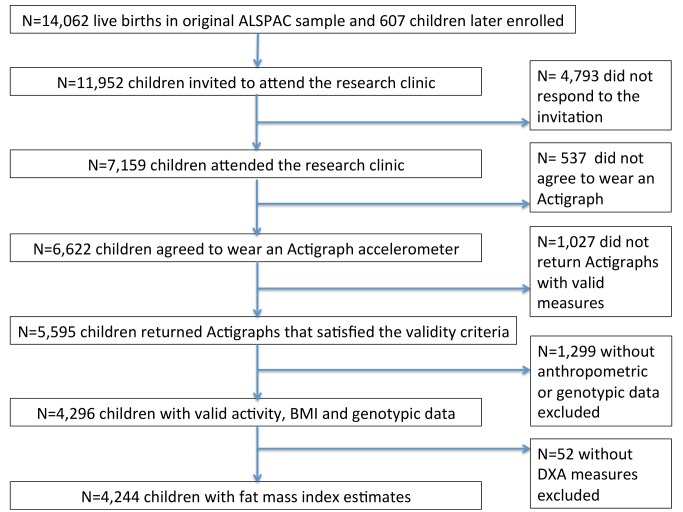
Participants in ALSPAC and in the analyses presented in this paper. DXA, dual energy X-ray absorptiometry.

Of the 11,952 children who were invited to attend the research clinic, 7,159 (59.9%) came to the clinic. 6,622 of the 7,159 (92.5%) agreed to wear an Actigraph accelerometer, and 5,595 of the 6,622 (84.5%) returned Actigraph accelerometer data that satisfied the validity criteria. Of the 5,595, BMI and genotypic data were available for 4,296 children (76.8%). FMI estimates were available for 4,244 children (75.8%) (Figure 2).

Of the individuals included in this analysis, 22.1% (950/4,296) were defined as being overweight and 4.2% (181/4,296) as obese, according to age- and sex-specific cutoffs proposed by the International Obesity Task Force [60]. A comparison of the baseline characteristics of individuals who did and did not attend the age 11-y clinic has been described in detail elsewhere [1]. Differences in baseline characteristics between the subset of children included in this analysis and those who did not attend the age 11-y clinic are shown in Table S3. The children included in this analysis were more likely to be girls and had a higher birth weight, higher gestational age at birth, higher social class, higher dietary intake at age 10 y and mothers who were less likely to be smokers and were more highly educated.

### Observational Analysis

From observational analysis of baseline characteristics, objectively assessed activity levels were higher for boys than girls for total physical activity (664.6 versus 555.1 mean movement counts/min, *p*<0.001) and for moderate-to-vigorous activity (25.8 versus 16.0 median min/d, *p*<0.001). Sedentary time was higher for girls than for boys (435.6 versus 418.2 mean min/d, *p*<0.001), as were mean values of BMI (19.1 versus 18.7 kg/m^2^, *p*<0.001) and fat mass (12.7 versus 10.2 kg, *p*<0.001) (Table 1). BMI and FMI were strongly correlated (Pearson's correlation coefficient = 0.94).

**Table 1:** Baseline characteristics of children.

Variable	All (*n* = 4,296)		Boys (*n* = 2,044)		Girls (*n* = 2,252)	
	Mean or Percent	SD	Mean or Percent	SD	Mean or Percent	SD
**Age (mo)**	140.8	2.8	140.8	2.8	140.8	2.7
**Height (cm)**	150.7	7.2	150.0	7.1	151.3	7.2
**Weight (kg)**	43.3	9.7	42.4	9.5	44.2	9.8
**BMI (kg/m^2^)**	18.9	3.3	18.7	3.2	19.1	3.4
**Fat mass (kg) measured by DXA**	11.5	6.5	10.2	6.4	12.7	6.4
**FMI (kg/m^2^)**	5.0	2.7	4.5	2.6	5.5	2.6
**Body fat percentage (fat mass [kg]/weight [kg])**	25.4	9.1	22.5	9.2	27.3	8.4
**Total physical activity (counts/min)**	607.2	178.4	664.6	187.9	555.1	151.7
**Moderate-to-vigorous-intensity physical activity (min/d)** a	20.0	12.0–31.4	25.8	15.9–38.5	16.0	9.9–24.9
**Sedentary time (min/d)**	427.3	66.6	418.2	68.5	435.6	63.7
**Birth weight (g)**	3,433.8	526.7	3,483.5	568.3	3,388.3	481.2
**Gestational age at birth (wk)**	39.5	1.8	39.4	1.9	39.6	1.6
**Maternal BMI (kg/m^2^)**	22.9	3.7	23.0	3.7	22.9	3.7
**Total daily dietary intake (kcal/d)**	1,862.0	377.3	1,953.5	389.8	1,778.3	344.9
**Maternal smoking during pregnancy**						
No	80.1%		80.1%		80.1%	
Yes	19.9%		19.9%		19.9%	
**Maternal education**						
Education up to age 16 y with certificate of secondary education or vocational training	20.0%		21.0%		19.1%	
Education up to age 16 y with general certificate of education (Ordinary level)	35.1%		34.4%		35.8%	
Education up to age 18 y with general certificate of education (Advanced level)	27.2%		26.8%		27.5%	
University degree	17.7%		17.8%		17.6%	
**Parental social class** b						
I Professional occupations	16.3%		16.5%		16.2%	
II Managerial and technical occupations	45.9%		45.5%		46.3%	
III(NM) Skilled non-manual occupations	24.4%		24.8%		24.0%	
III(M) Skilled manual occupations	9.6%		9.9%		9.4%	
IV Partly skilled occupations	3.3%		2.9%		3.6%	
V Unskilled occupations	0.5%		0.5%		0.5%	
**Stage of puberty** c						
Stage 1	47.7%		66.7%		33.2%	
Stage 2	35.0%		28.8%		39.8%	
Stage 3	13.7%		4.2%		21.0%	
Stage 4	3.1%		0.4%		5.2%	
Stage 5	0.5%		0%		0.8%	

Total sample sizes range from 3,121 to 4,098 depending on the availability of the data.

a Median and interquartile ranges are displayed for this variable because it is skewed.

b Based on parent with highest social class, as defined by the 1991 British Office of Population Censuses and Surveys classification.

c Based on highest Tanner scale developmental stage of breasts and pubic hair for females and pubic hair for males.

DXA, dual energy X-ray absorptiometry.

A 3.3 kg/m^2^ (1 SD) higher BMI was associated with 22.3 (95% CI, 17.0, 27.6) counts/min less total physical activity (*p* = 1.6×10^−16^), 2.6 (2.1, 3.1) min/d less moderate-to-vigorous activity (*p* = 3.7×10^−29^), and 3.5 (1.5, 5.5) min/d more sedentary time (*p* = 5.0×10^−4^). These associations were stronger when using FMI instead of BMI and were largely unaltered by adjusting for additional confounders (Table 2). In observational analyses stratified by sex, effect estimates were larger in boys for all activity phenotypes (Table S11).

**Table 2:** Associations between measures of adiposity and physical activity levels.

Adiposity	Activity	Model A^a^				Model B^b^			
		*N*	*Z*-Score Value Coefficient (95% CI)^c^	Difference in Activity, Raw Units (95% CI)^c^	*p*-Value	*N*	*Z*-Score Value Coefficient (95% CI)^c^	Difference in Activity, Raw Units (95% CI)^c^	*p*-Value
**BMI (kg/m^2^)**	Total physical activity (counts/min)	4,296	−0.12 (−0.15, −0.10)	−22.3 (−27.6, −17.0)	1.6×10^−16^	1,338	−0.13 (−0.20, −0.05)	−22.8 (−36.6, −8.7)	0.002
	Moderate-to-vigorous activity (min/d)^d^		−0.17 (−0.20, −0.14)	−2.6 (−3.1, −2.1)	3.7×10^−29^		−0.17 (−0.24, −0.09)	−2.6 (−3.7, −1.4)	1.8×10^−5^
	Sedentary time (min/d)		0.05 (0.02, 0.08)	3.5 (1.5, 5.5)	5.0×10^−4^		0.07 (−0.01, 0.15)	4.6 (−0.5, 9.7)	0.06
**FMI (kg/m^2^)**	Total physical activity (counts/min)	4,244	−0.18 (−0.21, −0.15)	−32.3 (−37.6, −27,1)	1.5×10^−33^	1,320	−0.22 (−0.29, −0.14)	−39.1 (−52.4, −25.8)	7.9×10^−9^
	Moderate-to-vigorous activity (min/d)^d^		−0.22 (−0.25, −0.19)	−3.4 (−3.8, −2.9)	2.4×10^−48^		−0.24 (−0.31, −0.17)	−3.7 (−4.8, −2.6)	4.0×10^−11^
	Sedentary time (min/d)		0.09 (0.06, 0.12)	5.8 (3.8, 7.7)	5.4×10^−6^		0.14 (0.07, 0.21)	9.5 (4.8, 14.2)	1.1×10^−4^

a Model A: adjusted for age.

b Model B: adjusted for age, birth weight, gestational age at birth, maternal smoking during pregnancy, maternal education, parental social class, maternal BMI, stage of puberty, total daily dietary intake, and intake of main food groups.

c Coefficients are displayed as sex-specific *z*-scores for both measures of adiposity and activity levels and have also been rescaled to give more meaningful outcomes relating to the raw units of these variables. The raw-unit difference was computed by multiplying the *z*-score value by the SD of the variable, taken from Table 1.

d Moderate-to-vigorous activity was log transformed for analysis.

### Direct Genotypic Associations

The BMI allelic score was normally distributed, with a mean of 29.6, SD of 3.9, and range of 16.3–42.3 (Figure S1). A per (average BMI-increasing) allele change in the allelic score was associated with a 0.14 (95% CI, 0.12, 0.17) kg/m^2^ increase in BMI (*p* = 5.5×10^−29^), and a 0.11 (0.09, 0.13) kg/m^2^ increase in FMI (*p* = 2.3×10^−25^) (Table 3). The BMI allelic score explained 2.8% of the variance in standardised BMI in this cohort, and 2.5% of the variance in FMI.

**Table 3:** Associations between the weighted allelic score for 32 SNPs and body mass index/fat mass index and activity measures.

Outcome	Per Allele Effects				Per Allele Effects (Adjusted for Activity^a^)		
	*N*	*Z*-Score Value Coefficient (95% CI)^b^	Difference in Adiposity or Activity, Raw Units (95% CI)^b^	*p*-Value	*Z*-Score Value Coefficient (95% CI)^b^	Difference in Adiposity or Activity, Raw Units (95% CI)^b^	*p*-Value
BMI (kg/m^2^)	4,296	0.04 (0.04, 0.05)	0.14 (0.12, 0.17)	5.5×10^−29^	0.04 (0.03, 0.05)	0.14 (0.11, 0.16)	4.2×10^−28^
FMI (kg/m^2^)	4,244	0.04 (0.03, 0.05)	0.11 (0.09, 0.13)	2.3×10^−25^	0.04 (0.03, 0.05)	0.10 (0.08, 0.12)	2.3×10^−24^
Total physical activity (counts/min)	4,296	−0.01 (0.00, −0.02)	−1.4 (−2.8, −0.03)	0.05			
Moderate-to-vigorous activity (min/d)^c^	4,296	−0.01 (−0.02, 0.00)	−0.12 (−0.24, −0.00)	0.05			
Sedentary time (min/d)	4,296	0.01 (0.00, 0.02)	0.57 (0.06, 1.1)	0.03			

Regression results were adjusted for age. Per (average BMI-increasing) allele effects were obtained by linear regression for all of these continuous outcome variables.

a Activity variables were total physical activity, moderate-to-vigorous and minutes of sedentary time.

b Coefficients are displayed as sex-specific *z*-scores for both measures of adiposity and activity levels and have also been rescaled to give more meaningful outcomes relating to the raw units of these variables. The raw-unit difference was computed by multiplying the *z*-score value by the SD of the variable, taken from Table 1.

c Moderate-to-vigorous activity was log transformed for analysis.

In contrast to BMI and FMI, confounding factors were not associated with the genotypes in this cohort (Table S4). Although the allelic score showed some weak associations with reported dietary intake and certain food groups and macronutrients (Table S5), these associations are largely driven by the inclusion of *FTO* in the score. As dietary intake is a known mediator in the association between *FTO* and adiposity [61], adjustment in instrumental variable analysis would not be appropriate.

A per allele change in the BMI allelic score was associated with a decrease of 1.4 (95% CI, 0.0, 2.8) counts/min of total physical activity (*p* = 0.05), an approximate decrease of 0.1 (0.0, 0.2) min/d of moderate-to-vigorous activity (*p* = 0.05), and an increase of 0.6 (0.1, 1.1) min/d of sedentary time (*p* = 0.03) (Table 3).

### Mendelian Randomization

Instrumental variable analysis using the BMI allelic score showed that a 3.3 kg/m^2^ higher BMI was associated with 32.4 (95% CI, 0.9, 63.9) counts/min less total physical activity (*p* = 0.04) (equivalent to 5.3% of the mean counts/min), 2.8 (0.1, 5.5) min/d less moderate-to-vigorous activity (*p* = 0.04), and 13.2 (1.3, 25.2) min/d more sedentary time (*p* = 0.03) (*F*-statistic = 124.9; partial *R* ^2^ = 0.03).

There was no evidence of a departure of instrumental-variable-derived estimates from observational results, as demonstrated by DWH tests (*p*≥0.10), indicating similarity between observational and MR estimates in the effect of BMI on physical activity levels. Furthermore, point estimates for effect sizes from the instrumental variable analysis were equal to or greater than those derived from basic observational analyses for all traits, though wider confidence intervals for the instrumental variable estimates resulted in larger *p*values.

Similar results were found when FMI was instrumented (Table 4). In addition, similar results were found when using physical activity and adiposity data for individuals at age 13 y, though the number of individuals at this time point was smaller (Table S6). In sensitivity analyses stratified by sex, wide confidence intervals for instrumental variable estimates did not allow the resolution of differences between boys and girls (Table S11).

**Table 4:** Associations between body mass index/fat mass index and activity levels as tested both by conventional epidemiological approaches and through the application of instrumental variable analysis using a 32-SNP weighted allelic score as an instrument.

Adiposity	Activity	*N*	Linear Regression			Instrumental Variable Regression (Weighted Allelic Score with 32 SNPs)					
			*Z*-Score Value Coefficient (95% CI)^a^	Difference in Activity, Raw Units (95% CI)^a^	*p*-Value	*F*-Statistic	Partial *R* ^2^	*Z*-Score Value Coefficient (95% CI)^a^	Difference in Activity, Raw Units (95% CI)^a^	*p*-Value	*p*-Value (DWH)^b^
**BMI (kg/m^2^)**	Total physical activity (counts/min)	4,296	−0.12 (−0.15, −0.10)	−22.3 (−27.6, −17.0)	1.6×10^−16^	124.85	0.03	−0.18 (−0.36, 0.00)	−32.4 (−63.9, −0.87)	0.04	0.52
	Moderate-to-vigorous activity (min/d)^c^		−0.17 (−0.20, −0.14)	−2.6 (−3.1, −2.1)	3.7×10^−29^			−0.18 (−0.36, −0.01)	−2.8 (−5.5, −0.08)	0.04	0.89
	Sedentary time (min/d)		0.05 (0.02, 0.08)	3.5 (1.5, 5.5)	5.0×10^−4^			0.20 (0.02, 0.38)	13.2 (1.3, 25.2)	0.03	0.10
**FMI (kg/m^2^)**	Total physical activity (counts/min)	4,244	−0.18 (−0.21, −0.15)	−32.3 (−37.6, −27,1)	1.5×10^−33^	108.34	0.03	−0.20 (−0.39, −0.02)	−36.1 (−69.3, −2.8)	0.03	0.82
	Moderate-to-vigorous activity (min/d)^c^		−0.22 (−0.25, −0.19)	−3.4 (−3.8, −2.9)	2.4×10^−48^			−0.20 (−0.39, −0.02)	3.1 (−5.9, −0.24)	0.03	0.86
	Sedentary time (min/d)		0.09 (0.06, 0.12)	5.8 (3.8, 7.7)	1.4×10^−8^			0.22 (0.03, 0.41)	14.6 (1.9, 27.3)	0.02	0.16

Regression results were adjusted for age.

a Coefficients are displayed as sex-specific *z*-scores for both measures of adiposity and activity levels and have also been rescaled to give more meaningful outcomes relating to the raw units of these variables. The raw-unit difference was computed by multiplying the *z*-score value by the SD of the variable, taken from Table 1.

b *p*(DWH) is the *p*-value of the Durbin form of the DWH test, which examines the difference between the estimates from linear regression and instrumental variable analysis.

c Moderate-to-vigorous activity was log transformed for analysis.

### Multiple Independent Instruments

**Figure 3:**
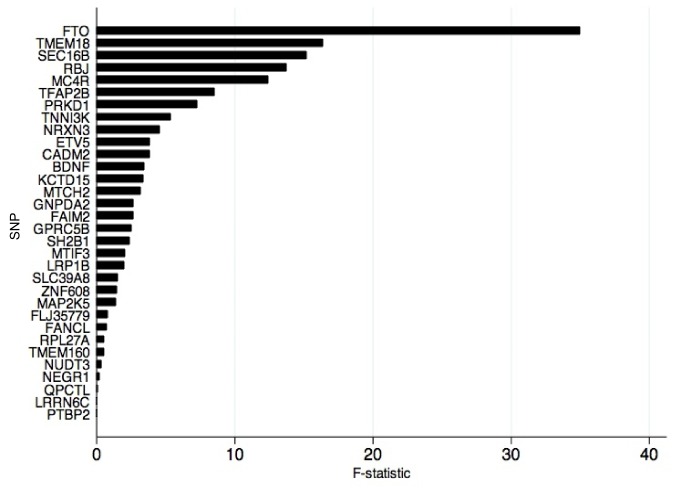
Strength of individual genetic variants for BMI as genetic instruments in instrumental variable analysis. *F*-statistic derived from first-stage regression in two-stage least squares analysis where each of the 32 alleles was used as an individual instrument for BMI.

An analysis of the alleles included in the BMI allelic score showed that rs1558902 (*FTO*) was the variant contributing most to its association with BMI (Figure 3). Results of instrumental variable analysis using this genetic variant were compared with those of a weighted allelic score consisting of the 31 genetic variants excluding *FTO* (Tables 5 and 6). The instrumented effect for *FTO* showed some difference to observational estimates, especially for sedentary time, where the instrumental variable analysis produced larger effect estimates than the observational analysis (*p* = 0.01 for DWH test). However, there was no strong statistical evidence that the instrumented effects of BMI on activity levels were different from one another (*p* for heterogeneity ≥0.06). An additional analysis was run that showed that independent pairs of variants from the 32 SNPs have normally distributed instrumental variable effects. Although pairs of variants including *FTO* lie at the lower end of this distribution, indicating that variation in *FTO* produces a larger-than-average effect in the instrumental variable analysis, this effect is not an outlier (Figure S2). Similar results were found when FMI was instrumented.

**Table 5:** Associations between body mass index/fat mass index and activity levels as tested both by conventional epidemiological approaches and through the application of instrumental variable analysis using the *FTO* (rs1558902) genetic variant as an instrument.

Adiposity	Activity	N	Linear Regression			Instrumental Variable Regression (*FTO* rs1558902)					
			*Z*-Score Value Coefficient (95% CI)^a^	Difference in Activity, Raw Units (95% CI)^a^	*p*-Value	*F*-Statistic	Partial *R* ^2^	*Z*-Score Value Coefficient (95% CI)^a^	Difference in Activity, Raw Units (95% CI)^a^	*p*-Value	*p*-Value (DWH)^b^
**BMI**	Total physical activity (counts/min)	4,296	−0.12 (−0.15, −0.10)	−22.3 (−27.6, −17.0)	1.6×10^−16^	35.0	0.01	−0.31 (−0.64, −0.03)	−54.5 (−114.3, 5.3)	0.07	0.28
	Moderate-to-vigorous activity (min/d)^c^		−0.17 (−0.20, −0.14)	−2.6 (−3.1, −2.1)	3.7×10^−29^			−0.16 (−0.49, 0.17)	−2.4 (−7.5, 2.6)	0.34	0.95
	Sedentary time (min/d)		0.05 (0.02, 0.08)	3.5 (1.5, 5.5)	5.0×10^−4^			0.50 (0.13, 0.86)	33.0 (8.9, 57.2)	0.007	0.01
**FMI**	Total physical activity (counts/min)	4,244	−0.18 (−0.21, −0.15)	−32.3 (−37.6, −27,1)	1.5×10^−33^	34.7	0.01	−0.31 (−0.64, 0.02)	−55.3 (−114.1, 3.4)	0.07	0.44
	Moderate-to-vigorous activity (min/d)^c^		−0.22 (−0.25, −0.19)	−3.4 (−3.8, −2.9)	2.4×10^−48^			−0.16 (−0.48, 0.17)	−2.4 (−7.4, 2.6)	0.35	0.71
	Sedentary time (min/d)		0.09 (0.06, 0.12)	5.8 (3.8, 7.7)	1.4×10^−8^			0.50 (0.14, 0.86)	33.4 (9.5, 57.3)	0.006	0.01

Regression results were adjusted for age.

a Coefficients are displayed as sex-specific *z*-scores for both measures of adiposity and activity levels and have also been rescaled to give more meaningful outcomes relating to the raw units of these variables. The raw-unit difference was computed by multiplying the *z*-score value by the SD of the variable, taken from Table 1.

b *p*(DWH) is the *p*-value of the Durbin form of the DWH test, which examines the difference between the estimates from linear regression and instrumental variable analysis.

c Moderate-to-vigorous activity was log transformed for analysis.

**Table 6:** Associations between body mass index/fat mass index and activity levels as tested both by conventional epidemiological approaches and through the application of instrumental variable analysis using a 31-SNP weighted allelic score (excluding *FTO*) as an instrument.

Adiposity	Activity	*N*	Linear Regression			Instrumental Variable Regression (Weighted Allelic Score with 31 SNPs)					
			*Z*-Score Value Coefficient (95% CI)^a^	Difference in Activity, Raw Units (95% CI)^a^	*p*-Value	*F*-Statistic	Partial *R* ^2^	*Z*-Score Value Coefficient (95% CI)^a^	Difference in Activity, Raw Units (95% CI)^a^	*p*-Value	*p*-Value (DWH)^b^
BMI	Total physical activity (counts/min)	4,296	−0.12 (−0.15, −0.10)	−22.3 (−27.6, −17.0)	1.6×10^−16^	88.9	0.02	−0.13 (−0.34, 0.07)	−24.0 (−61.2, 13.1)	0.21	0.92
	Moderate-to-vigorous activity (min/d)^c^		−0.17 (−0.20, −0.14)	−2.6 (−3.1, −2.1)	3.7×10^−29^			−0.19 (−0.40, 0.02)	−2.9 (−6.1, 0.3)	0.07	0.85
	Sedentary time (min/d)		0.05 (0.02, 0.08)	3.5 (1.5, 5.5)	5.0×10^−4^			0.09 (−0.12, 0.30)	5.8 (−8.2, 19.7)	0.42	0.75
FMI	Total physical activity (counts/min)	4,244	−0.18 (−0.21, −0.15)	−32.3 (−37.6, −27,1)	1.5×10^−33^	73.3	0.02	−0.16 (−0.38, 0.07)	−28.1 (−68.4, 12.2)	0.17	0.84
	Moderate-to-vigorous activity (min/d)^c^		−0.22 (−0.25, −0.19)	−3.4 (−3.8, −2.9)	2.4×10^−48^			−0.22 (−0.44, 0.01)	−3.4 (−6.8, 0.8)	0.06	0.99
	Sedentary time (min/d)		0.09 (0.06, 0.12)	5.8 (3.8, 7.7)	1.4×10^−8^			0.10 (−0.13, 0.33)	6.8 (−8.4, 22.1)	0.38	0.89

Regression results were adjusted for age.

a Coefficients are displayed as sex-specific *z*-scores for both measures of adiposity and activity levels and have been rescaled to give more meaningful outcomes relating to the raw units of these variables. The raw-unit difference was computed by multiplying the *z*-score value by the SD of the variable, taken from Table 1.

b *p*(DWH) is the *p*-value of the Durbin form of the DWH test, which examines the difference between the estimates from linear regression and instrumental variable analysis.

c Moderate-to-vigorous activity was log transformed for analysis.

### Genome-Wide Prediction for Physical Activity

The additive heritability of activity measures was estimated to be 17%–25% (Table S2), indicating a non-negligible contribution of genetic variation to variance in physical activity levels. Genome-wide prediction scores were generated for each of the activity measures and applied in instrumental variable analysis to independent subgroups. Physical activity scores were normally distributed and showed some association with their respective activity measures in the other subgroups (Table S7). The physical activity scores had no substantive correlation with the BMI allelic score (Table S8), providing evidence that the instruments for adiposity and physical activity were independent of each other.

There was no strong statistical evidence that the instrumented effects of activity on adiposity in the subgroups were different from each other (*p* for heterogeneity ≥0.11). A meta-analysis of both instrumental variable analyses, unlike observational analysis, found no strong evidence for a causal effect of physical activity on BMI at age 11 y (regression coefficient 0.27 [95% CI, −0.41, 0.94], *p* = 0.44, for total physical activity; −0.03 [−0.72, 0.66], *p* = 0.93, for moderate-to-vigorous activity; −0.51 [−1.24, 0.22], *p* = 0.17, for sedentary time, with *z*-standardised units of BMI and activity measures) (Table S9). Results were similar when FMI was the outcome (Table S10). However, confidence intervals were wide, and small *F*-statistics indicated that caution should be applied when using these instruments for physical activity (*F*≤6.80).

## Discussion

This study used a MR approach to investigate a causal role for elevated BMI and FMI in lower physical activity levels in children. In agreement with previous findings that adiposity loci identified by GWASs in adults are associated with childhood anthropometric traits [62],[63], the allelic score derived from established genetic variants for BMI was strongly associated with both exposures of interest (BMI and FMI). The allelic score explained a larger proportion of the variation in BMI compared with FMI. Nonetheless, the *F*-statistic for the association between the allelic score and FMI was large (>100), supporting previous findings for consistent associations between the 32 BMI-associated loci and other measures of adiposity [64].

The similarity between observational and instrumental variable estimates for the association between BMI and physical activity provides evidence suggesting that increasing adiposity leads to a causal reduction in total and moderate-to-vigorous physical activity, and a causal increase in the length of sedentary time. Associations from instrumental variable analysis were marginally stronger between FMI and activity levels, as expected given that BMI does not differentiate between fat and lean mass, and lean mass correlates positively with levels of activity. These results are in line with findings from recent prospective studies that show that fatness is predictive of reduced physical activity at later time points [7],[20],[22].

The finding that the calculated effect sizes in this MR analysis account for a substantial proportion of the association between adiposity and activity identified in observational studies [1]–[6] has important public health implications. Whilst the mechanisms of this pathway are unclear and may constitute both physiological and psychological factors [65],[66], evidence that adiposity is a causal risk factor for low physical activity is important, since it has been recommended that children spend at least 60 min in moderate-to-vigorous-intensity physical activity each day in order to maintain their physical health [67]–[73]. In particular, this evidence highlights the importance of developing programmes targeting body weight in order to increase physical activity levels in overweight children [74].

A limitation of the study was that we were not able to collect physical activity, body composition, or genetic data on a substantial number of children originally enrolled in the study. These missing data can lead to a bias if the causal effect of adiposity on physical activity (and vice versa) is different in the children who did not take part. Whilst we cannot fully exclude such a bias, associations were not altered by adjustment for factors associated with missing data [1]. Possible limitations to the MR analysis in general include the possibility of population stratification, canalization, power deficiency, pleiotropy, and linkage disequilibrium [23],[47]. Major population stratification is unlikely since this analysis was completed in unrelated individuals of European ancestry. A pleiotropic association of a genetic variant included in the allelic score with the outcome, or linkage disequilibrium with a functional variant associated with the outcome, would violate the assumptions of MR analysis. Multiple independent instruments were used to provide evidence against the existence of shared pleiotropy and against the influence of linkage-disequilibrium-induced confounding [48]. Whereas instrumental variable estimates for the association between adiposity and physical activity obtained using the 31-SNP allelic score (excluding *FTO*) were consistently similar to observational effect sizes, the estimates produced using the *FTO* variant as an instrument were generally larger than observational findings. However, there was no strong statistical evidence for a difference between the instrumented estimates, arguing against a pleiotropic effect. It should be emphasised that this investigation does not provide definitive evidence against the existence or impact of pleiotropy, and more functional knowledge of the variants is required to assess this more comprehensively.

With evidence for causality in the direction from adiposity to activity levels, further analyses were undertaken to address the reciprocal association between physical activity and levels of adiposity in children at age 11 y. The absence of a causal effect of physical activity on adiposity goes some way towards explaining the lack of impact of short-term physical activity intervention trials on adiposity levels in children [11]–[13]. It is also in line with the fact that there is little evidence that there has been a major decline in physical activity during the course of the obesity epidemic [75], compared with stronger evidence that there has been an increase in energy intake in the same time period [76]. Although no causal effect was shown in our preliminary analysis, this analysis is likely to suffer from limitations of small sample sizes and inadequacy of the prediction scores for physical activity in terms of the association between genotype and physical activity [77]–[79], genetic confounding, or pleiotropy. In addition, split sample instrumental variable methods have been shown to generate estimates that are biased towards the null [58]. Before we can confirm or refute a complete lack of effect of activity levels on adiposity using MR analysis, a well-powered study with strong genetic instruments for physical activity variables is required. Therefore, findings from this secondary analysis do not exclude lower physical activity also leading to increases in adiposity and a “vicious cycle” being initiated [9].

Results of our main analysis suggest that increased adiposity leads to a reduction in physical activity. Although further work is required to determine a more accurate estimate of the causal effect in the reverse direction, this study provides insight into the causal contributions of adiposity to activity levels in children and supports research into the targeting of BMI in efforts to increase childhood activity levels.

## Supporting Information

Figure S1 **Distribution of the BMI allelic score in this study population.**
(TIF)

Figure S2 **Distribution of** ***z*** **-statistics for pair combinations of the 32 SNPs in instrumental variable regressions.** Blue indicates the distribution of *z*-statistics for all pair combinations of the 32 SNPs; red indicates the distribution of *z*-statistics for combinations of the 32 SNPs where one SNP was the *FTO* (rs1558902) variant. Median coefficient using pair combinations of the 32 SNPs in instrumental variable regression = −0.13 SD counts/min per 1-SD increase in BMI. Coefficient using 32-SNP score in instrumental variable regression = −0.18 SD counts/min per 1-SD increase in BMI. Coefficient from observational regression = −0.12 SD counts/min per 1-SD increase in BMI.
(TIF)

Table S1 **Independent genetic variants from a meta-analysis of GWASs for BMI included in weighted allelic score.** *[33]. ^±^this value ranges from 0 to 1 and indicates the squared correlation between imputed and true genotypes. ^$^SD change in sex- and age-specific BMI per allele increase.
(DOCX)

Table S2 **GCTA analysis—physical activity trait variance explained by SNPs.** h^2^, additive heritability, or variance explained by all 500,527 SNPs in the genotypic data for physical activity measures in children, estimated using a restricted maximum likelihood method. *p*-Value is for a test that the additive heritability calculated is non-zero. *Moderate-to-vigorous physical activity was log transformed for analysis.
(DOCX)

Table S3 **Comparison of baseline characteristics between children included in this analysis compared with individuals who did not attend the age 11-y research clinic.** *Total sample size varies depending on the availability of the data. ^a^Based on partner with highest social class. ^b^Based on highest Tanner scale developmental stage of breasts and pubic hair for females and pubic hair for males. ^$^Data on individuals in core ALSPAC cohort originally recruited and children later enrolled.
(DOCX)

Table S4 **Associations between body mass index, fat mass index, and genotypes and possible confounding factors.** Coef, beta coefficient; OR, odds ratio. Per SD effects were obtained for the confounding variables by linear regression with BMI/FMI. Per allele effects were obtained by linear regression with the allelic scores and *FTO* genotype. Maternal smoking during pregnancy was the only binary outcome variable, and so logistic regression was used to obtain odds ratios for the per SD effects of BMI/FMI and the per allele effects of allelic scores and *FTO* genotype. Effects were adjusted for age, and also for BMI in regressions involving genotype. *Sample size varies from 3,121 to 4,098 depending on completeness of data on confounding factors.
(DOCX)

Table S5 **Associations between body mass index, fat mass index, and genotypes and components of dietary intake.** Coef, beta coefficient; OR, odds ratio. Per SD effect sizes were obtained for the confounding variables by linear regression with BMI/FMI. Per allele effects were obtained by linear regression with the allelic scores and *FTO* genotype. Effects were adjusted for age, and also for BMI in regressions involving genotype. *Sample size varies from 2,103 to 3,991 depending on completeness of data on confounding factors. ^±^Excluding dietary under-reporters.
(DOCX)

Table S6 **Associations between body mass index/fat mass index and activity levels as tested both by conventional epidemiological approaches and through the application of instrumental variable analysis using a 32-SNP weighted allelic score as an instrument at age 13 y.** Regression results were adjusted for age. Coefficients are displayed as sex-specific *z*-scores for both measures of adiposity and activity levels. P(DWH) is the *p*-value of the Durbin form of the DWH test, which examines the difference between the estimates from linear regression and instrumental variable analysis. *Moderate-to-vigorous activity was log transformed for analysis.
(DOCX)

Table S7 **Associations between genome-wide prediction scores and activity measures in independent subgroups.** Regression results were adjusted for age. Per allele effects were obtained by linear regression for all of these continuous variables. Coefficients are based on *z*-scores for activity levels. *Moderate-to-vigorous activity was log transformed for analysis.
(DOCX)

Table S8 **Correlations between genome-wide prediction scores and the BMI allelic score.** Pearson product-moment correlation coefficients calculated.
(DOCX)

Table S9 **Associations between activity levels and body mass index as tested both by conventional epidemiological approaches and through the application of instrumental variable analysis using genome-wide prediction scores for activity levels: meta-analysis for two sets of prediction scores.** Regression results were adjusted for age. Coefficients are based on *z*-scores for activity and adiposity levels. P(DWH) is the *p*-value of the Durbin form of the DWH test, which examines the difference between the estimates from linear regression and instrumental variable analysis. *Moderate-to-vigorous activity was log transformed for analysis. ^$^Physical activity prediction scores were generated in one subgroup and applied to individuals in a second independent subgroup for instrumental variable analysis.
(DOCX)

Table S10 **Associations between activity levels and fat mass index as tested both by conventional epidemiological approaches and through the application of instrumental variable analysis using genome-wide prediction scores for activity levels: meta-analysis for two sets of prediction scores.** Regression results were adjusted for age. Coefficients are based on *z*-scores for activity and adiposity levels. P(DWH) is the *p*-value of the Durbin form of the DWH test, which examines the difference between the estimates from linear regression and instrumental variable analysis. *Moderate-to-vigorous activity was log transformed for analysis. ^$^Physical activity prediction scores were generated in one subgroup and applied to individuals in a second independent subgroup for instrumental variable analysis.
(DOCX)

Table S11 **Associations between body mass index/fat mass index and activity levels as tested both by conventional epidemiological approaches and through the application of instrumental variable analysis using a 32-SNP weighted allelic score as an instrument: analysis stratified by sex.** Regression results were adjusted for age. Coefficients are displayed as sex-specific *z*-scores for both measures of adiposity and activity levels. P(DWH) is the *p*-value of the Durbin form of the DWH test, which examines the difference between the estimates from linear regression and instrumental variable analysis. *Moderate-to-vigorous activity was log transformed for analysis.
(DOCX)

Table S12 **Associations between activity levels and body mass index as tested both by conventional epidemiological approaches and through the application of instrumental variable analysis using genome-wide prediction scores for activity levels: analysis stratified by sex.** Regression results were adjusted for age. Coefficients are based on *z*-scores for activity and adiposity levels. P(DWH) is the *p*-value of the Durbin form of the DWH test, which examines the difference between the estimates from linear regression and instrumental variable analysis. *Moderate-to-vigorous activity was log transformed for analysis. ^$^Physical activity prediction scores were generated in one subgroup and applied to individuals in a second independent subgroup for instrumental variable analysis.
(DOCX)

Table S13 **Associations between activity levels and fat mass index as tested both by conventional epidemiological approaches and through the application of instrumental variable analysis using genome-wide prediction scores for activity levels: meta-analysis for two sets of prediction scores stratified by sex.** Regression results were adjusted for age. P(DWH) is the *p*-value of the Durbin form of the DWH test, which examines the difference between the estimates from linear regression and instrumental variable analysis. *Moderate-to-vigorous activity was log transformed for analysis. ^$^Physical activity prediction scores were generated in one subgroup and applied to individuals in a second independent subgroup for instrumental variable analysis.
(DOCX)

## Abbreviations

ALSPAC: Avon Longitudinal Study of Parents and Children
BMI: body mass index
DWH: Durbin-Wu-Hausman
FMI: fat mass index
GWAS: genome wide association study
MR: Mendelian randomization
SD: standard deviation
SNP: single nucleotide polymorphism
