# Supplementary figures and images for: Assessing Causality in the Association between Child Adiposity and Physical Activity Levels: A Mendelian Randomization Analysis
Source: PLoS Med. 2014 Mar 18;11(3):e1001618. doi: 10.1371/journal.pmed.1001618 (PMC3958348; doi:10.1371/journal.pmed.1001618)

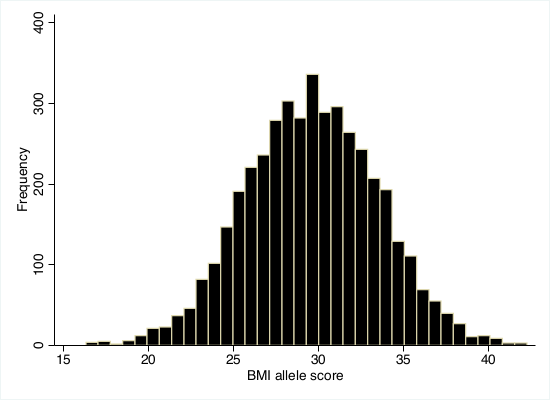

Supplement: Figure S1 — Distribution of the BMI allelic score in this study population. (TIF) [file pmed.1001618.s001.tif]

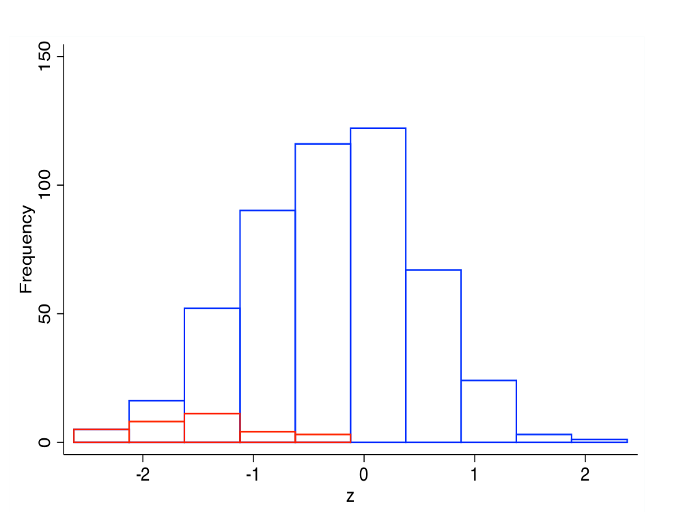

Supplement: Figure S2 — Distribution of z -statistics for pair combinations of the 32 SNPs in instrumental variable regressions. Blue indicates the distribution of z-statistics for all pair combinations of the 32 SNPs; red indicates the distribution of z-statistics for combinations of the 32 SNPs where one SNP was the FTO (rs1558902) variant. Median coefficient using pair combinations of the 32 SNPs in instrumental variable regression = −0.13 SD counts/min per 1-SD increase in BMI. Coefficient using 32-SNP score in instrumental variable regression = −0.18 SD counts/min per 1-SD increase in BMI. Coefficient from observational regression = −0.12 SD counts/min per 1-SD increase in BMI. (TIF) [file pmed.1001618.s002.tif]
