# Supplementary material for: Assessing Causality in the Association between Child Adiposity and Physical Activity Levels: A Mendelian Randomization Analysis
Source: PLoS Med. 2014 Mar 18;11(3):e1001618. doi: 10.1371/journal.pmed.1001618 (PMC3958348; doi:10.1371/journal.pmed.1001618)
Supplement: Table S1 — Independent genetic variants from a meta-analysis of GWASs for BMI included in weighted allelic score. *[33]. ±this value ranges from 0 to 1 and indicates the squared correlation between imputed and true genotypes. $SD change in sex- and age-specific BMI per allele increase. (DOCX) [file pmed.1001618.s003.docx]

| **SNP** | **Nearest Gene** | **Chromosome** | **Base pair position** | **Coefficient from GWAS* for weighting** | **Imputation quality in ALSPAC**  **(Rsq value) ^±^** | **BMI increasing allele/other allele** | **BMI increasing allele frequency in ALSPAC** | **Minor allele frequency in ALSPAC** | **Effect size in ALSPAC**^$^ | **Standard error in ALSPAC** |
| --- | --- | --- | --- | --- | --- | --- | --- | --- | --- | --- |
| rs10150332 | *NRXN3* | 14 | 79,006,717 | 0.13 | 0.9963 | C/T | 20.8% | 20.8% | 0.06 | 0.03 |
| rs10767664 | *BDNF* | 11 | 27,682,562 | 0.19 | 0.9965 | A/T | 80.0% | 20.0% | 0.05 | 0.03 |
| rs10938397 | *GNPDA2* | 4 | 44,877,284 | 0.18 | 0.9881 | G/A | 43.2% | 43.2% | 0.04 | 0.02 |
| rs10968576 | *LRRN6C* | 9 | 28,404,339 | 0.11 | 0.9995 | G/A | 31.6% | 31.6% | -0.01 | 0.02 |
| rs11847697 | *PRKD1* | 14 | 29,584,863 | 0.17 | 0.9688 | T/C | 4.7% | 4.7% | 0.14 | 0.05 |
| rs12444979 | *GPRC5B* | 16 | 19,841,101 | 0.17 | 0.9975 | C/T | 86.1% | 13.9% | 0.05 | 0.03 |
| rs13078807 | *CADM2* | 3 | 85,966,840 | 0.10 | 0.9966 | G/A | 19.7% | 19.7% | 0.05 | 0.03 |
| rs13107325 | *SLC39A8* | 4 | 103,407,732 | 0.19 | 0.9972 | T/C | 7.1% | 7.1% | 0.05 | 0.04 |
| rs1514175 | *TNNI3K* | 1 | 74,764,232 | 0.07 | 0.9984 | A/G | 42.2% | 42.2% | 0.05 | 0.02 |
| rs1555543 | *PTBP2* | 1 | 96,717,385 | 0.06 | 0.9960 | C/A | 59.3% | 40.7% | 0.00 | 0.02 |
| rs1558902 | *FTO* | 16 | 52,361,075 | 0.39 | 0.9967 | A/T | 41.1% | 41.1% | 0.13 | 0.02 |
| rs206936 | *NUDT3* | 6 | 34,410,847 | 0.06 | 0.9875 | G/A | 19.5% | 19.5% | -0.02 | 0.03 |
| rs2112347 | *FLJ35779* | 5 | 75,050,998 | 0.10 | 0.9945 | T/G | 63.9% | 36.1% | 0.02 | 0.02 |
| rs2241423 | *MAP2K5* | 15 | 65,873,892 | 0.13 | 0.9997 | G/A | 78.4% | 21.6% | 0.03 | 0.03 |
| rs2287019 | *QPCTL* | 19 | 50,894,012 | 0.15 | 0.9991 | C/T | 81.0% | 19.0% | -0.01 | 0.03 |
| rs2815752 | *NEGR1* | 1 | 72,585,028 | 0.13 | 0.9964 | A/G | 59.7% | 40.3% | 0.01 | 0.02 |
| rs2867125 | *TMEM18* | 2 | 612,827 | 0.31 | 0.9997 | C/T | 82.9% | 17.1% | 0.12 | 0.03 |
| rs2890652 | *LRP1B* | 2 | 142,676,401 | 0.09 | 0.9888 | C/T | 16.4% | 16.4% | 0.04 | 0.03 |
| rs29941 | *KCTD15* | 19 | 39,001,372 | 0.06 | 0.9999 | G/A | 68.7% | 31.3% | 0.04 | 0.02 |
| rs3810291 | *TMEM160* | 19 | 52,260,843 | 0.09 | 0.7652 | A/G | 68.9% | 31.1% | 0.02 | 0.03 |
| rs3817334 | *MTCH2* | 11 | 47,607,569 | 0.06 | 0.9984 | T/C | 40.2% | 40.2% | 0.04 | 0.02 |
| rs4771122 | *MTIF3* | 13 | 26,918,180 | 0.09 | 0.9313 | G/A | 23.5% | 23.5% | 0.04 | 0.03 |
| rs4836133 | *ZNF608* | 5 | 124,360,002 | 0.07 | 0.9429 | A/C | 49.0% | 49.0% | -0.03 | 0.02 |
| rs4929949 | *RPL27A* | 11 | 8,561,169 | 0.06 | 0.9671 | C/T | 54.9% | 45.1% | 0.02 | 0.02 |
| rs543874 | *SEC16B* | 1 | 176,156,103 | 0.22 | 0.9965 | G/A | 21.2% | 21.2% | 0.10 | 0.03 |
| rs571312 | *MC4R* | 18 | 55,990,749 | 0.23 | 0.9995 | A/C | 23.2% | 23.2% | 0.09 | 0.03 |
| rs713586 | *RBJ* | 2 | 25,011,512 | 0.14 | 0.9993 | C/T | 49.0% | 49.0% | 0.08 | 0.02 |
| rs7138803 | *FAIM2* | 12 | 48,533,735 | 0.12 | 0.9980 | A/G | 35.6% | 35.6% | 0.04 | 0.02 |
| rs7359397 | *SH2B1* | 16 | 28,793,160 | 0.15 | 0.9988 | T/C | 40.7% | 40.7% | 0.03 | 0.02 |
| rs887912 | *FANCL* | 2 | 59,156,381 | 0.10 | 0.9972 | T/C | 28.7% | 28.7% | -0.02 | 0.02 |
| rs9816226 | *ETV5* | 3 | 187,317,193 | 0.14 | 0.9556 | T/A | 82.6% | 17.4% | 0.06 | 0.03 |
| rs987237 | *TFAP2B* | 6 | 50,911,009 | 0.13 | 0.9994 | G/A | 17.3% | 17.3% | 0.08 | 0.03 |
