# Supplementary material for: Assessing Causality in the Association between Child Adiposity and Physical Activity Levels: A Mendelian Randomization Analysis
Source: PLoS Med. 2014 Mar 18;11(3):e1001618. doi: 10.1371/journal.pmed.1001618 (PMC3958348; doi:10.1371/journal.pmed.1001618)
Supplement: Table S2 — GCTA analysis—physical activity trait variance explained by SNPs. h2, additive heritability, or variance explained by all 500,527 SNPs in the genotypic data for physical activity measures in children, estimated using a restricted maximum likelihood method. p-Value is for a test that the additive heritability calculated is non-zero. *Moderate-to-vigorous physical activity was log transformed for analysis. (DOCX) [file pmed.1001618.s004.docx]

| **Variable** | **h**^2^ | **SE** | **P-value** |
| --- | --- | --- | --- |
| Total physical activity | 0.21 | 0.10 | 0.01 |
| MVPA* | 0.17 | 0.09 | 0.04 |
| Sedentary | 0.25 | 0.09 | 0.004 |
