# Supplementary material for: Assessing Causality in the Association between Child Adiposity and Physical Activity Levels: A Mendelian Randomization Analysis
Source: PLoS Med. 2014 Mar 18;11(3):e1001618. doi: 10.1371/journal.pmed.1001618 (PMC3958348; doi:10.1371/journal.pmed.1001618)
Supplement: Table S3 — Comparison of baseline characteristics between children included in this analysis compared with individuals who did not attend the age 11-y research clinic. *Total sample size varies depending on the availability of the data. aBased on partner with highest social class. bBased on highest Tanner scale developmental stage of breasts and pubic hair for females and pubic hair for males. $Data on individuals in core ALSPAC cohort originally recruited and children later enrolled. (DOCX) [file pmed.1001618.s005.docx]

| **Variable*** | **Individuals included in analysis (n=4,296)** | | **Individuals who did not attend research clinic (n= 7,510**^$^**)** | | **P-value for difference** |
| --- | --- | --- | --- | --- | --- |
|  | **Mean** | **SD** | **Mean** | **SD** |  |
| **Birth weight (g)** | 3433.8 | 526.7 | 3360.3 | 594.0 | <0.001 |
| **Gestational age (weeks)** | 39.5 | 1.8 | 39.3 | 2.2 | <0.001 |
| **Maternal BMI (kg/m^2^)** | 22.9 | 3.7 | 22.9 | 3.9 | 0.90 |
| **Total daily dietary intake at age 10 (kcal/day)** | 1862.0 | 377.3 | 1815.8 | 414.9 | 0.001 |
|  | **%** | | **%** | |  |
| **Sex** |  | |  | | <0.001 |
| Boys | 47.6 | | 53.8 | |  |
| Girls | 52.4 | | 46.2 | |  |
| **Smoking during pregnancy** | | |  | | <0.001 |
| No | 80.1 | | 58.6 | |  |
| Yes | 19.9 | | 41.3 | |  |
| **Maternal education** | | |  | | <0.001 |
| Education up to 16 with certificate of secondary education (CSE) or vocational training | 20.0 | | 39.0 | |  |
| Education up to 16 with general certificate of education (GCE/O-level) | 35.1 | | 33.8 | |  |
| Education up to 18 with general certificate of education (Advanced level) | 27.2 | | 17.7 | |  |
| University degree | 17.7 | | 9.5 | |  |
| **Parental social class ^a^** | | |  | | <0.001 |
| I Professional occupations | 16.3 | | 13.3 | |  |
| II Managerial and technical occupations | 45.9 | | 41.8 | |  |
| III(NM) Skilled non-manual occupations | 24.4 | | 25.5 | |  |
| III(M) Skilled manual occupations | 9.6 | | 13.5 | |  |
| IV Partly-skilled occupations | 3.3 | | 5.0 | |  |
| V Unskilled occupations | 0.5 | | 0.9 | |  |
| **Stage of puberty ^b^** | | |  | | 0.111 |
| Stage 1 | 47.7 | | 51.7 | |  |
| Stage 2 | 35.0 | | 31.1 | |  |
| Stage 3 | 13.7 | | 13.2 | |  |
| Stage 4 | 3.1 | | 3.4 | |  |
| Stage 5 | 0.5 | | 0.6 | |  |
