# Supplementary material for: Assessing Causality in the Association between Child Adiposity and Physical Activity Levels: A Mendelian Randomization Analysis
Source: PLoS Med. 2014 Mar 18;11(3):e1001618. doi: 10.1371/journal.pmed.1001618 (PMC3958348; doi:10.1371/journal.pmed.1001618)
Supplement: Table S4 — Associations between body mass index, fat mass index, and genotypes and possible confounding factors. Coef, beta coefficient; OR, odds ratio. Per SD effects were obtained for the confounding variables by linear regression with BMI/FMI. Per allele effects were obtained by linear regression with the allelic scores and FTO genotype. Maternal smoking during pregnancy was the only binary outcome variable, and so logistic regression was used to obtain odds ratios for the per SD effects of BMI/FMI and the per allele effects of allelic scores and FTO genotype. Effects were adjusted for age, and also for BMI in regressions involving genotype. *Sample size varies from 3,121 to 4,098 depending on completeness of data on confounding factors. (DOCX) [file pmed.1001618.s006.docx]

| **Variable*** | **Body mass index (kg/m**^2^**)** | | | **Fat mass index (kg/m**^2^**)** | | | **Weighted allelic score with 32 SNPs** | | | **Weighted allelic score with 31 SNPs** | | | **FTO genotype** | | |
| --- | --- | --- | --- | --- | --- | --- | --- | --- | --- | --- | --- | --- | --- | --- | --- |
|  | **Coef** | **95%CI** | **P** | **Coef** | **95%CI** | **P** | **Coef** | **95%CI** | **P** | **Coef** | **95%CI** | **P** | **Coef** | **95%CI** | **P** |
| Mother’s highest level of education | -0.10 | -0.14,-0.06 | 1.3x10^-7^ | -0.10 | -0.14,-0.07 | 9.4x10^-8^ | 0.01 | 0.00,0.01 | 0.30 | 0.00 | -0.01,0.01 | 0.68 | 0.04 | -0.02,0.09 | 0.18 |
| Parental social class | -0.03 | -0.07,0.00 | 0.05 | -0.03 | -0.07,0.00 | 0.07 | 0.00 | -0.01,0.01 | 0.68 | 0.00 | -0.01,0.01 | 0.49 | -0.01 | -0.06,0.04 | 0.73 |
| Mother’s pre-pregnancy BMI (kg/m^2^) | 1.31 | 1.20,1.42 | 5.0x10^-116^ | 1.23 | 1.12,1.36 | 1.8x10^-100^ | 0.00 | -0.03,0.03 | 0.90 | 0.00 | -0.03,0.03 | 0.86 | 0.00 | -0.16,0.15 | 0.95 |
| Gestational age (weeks) | 0.00 | -0.05,0.06 | 0.97 | -0.03 | -0.08,0.03 | 0.33 | 0.01 | 0.00,0.02 | 0.17 | 0.00 | -0.01,0.02 | 0.64 | 0.08 | 0.00,0.15 | 0.06 |
| Birthweight  (g) | 55.4 | 39.2,71.6 | 2.1x10^-11^ | 39.4 | 23.0,55.7 | 2.4x10^-6^ | 0.56 | -3.65,4.80 | 0.80 | 0.07 | -4.53,4.67 | 0.98 | 5.33 | -17.8,28.5 | 0.65 |
| Stage of puberty | 0.23 | 0.21,0.26 | 1.7x10^-68^ | 0.18 | 0.15,0.20 | 7.7x10^-39^ | 0.01 | 0.01,0.02 | 0.26 | 0.00 | -0.01,0.01 | 0.78 | 0.03 | 0.00,0.07 | 0.08 |
|  | **OR** | **95%CI** | **P** | **OR** | **95%CI** | **P** | **OR** | **95%CI** | **P** | **OR** | **95%CI** | **P** | **OR** | **95%CI** | **P** |
| Smoking during pregnancy (yes/no) | 1.23 | 1.13,1.33 | 4.7x10^-7^ | 1.20 | 1.10,1.30 | 7.8x10^-6^ | 1.00 | 0.98,1.02 | 0.93 | 1.00 | 0.98,1.02 | 0.89 | 1.00 | 0.89,1.13 | 0.95 |
