# Supplementary material for: Assessing Causality in the Association between Child Adiposity and Physical Activity Levels: A Mendelian Randomization Analysis
Source: PLoS Med. 2014 Mar 18;11(3):e1001618. doi: 10.1371/journal.pmed.1001618 (PMC3958348; doi:10.1371/journal.pmed.1001618)
Supplement: Table S5 — Associations between body mass index, fat mass index, and genotypes and components of dietary intake. Coef, beta coefficient; OR, odds ratio. Per SD effect sizes were obtained for the confounding variables by linear regression with BMI/FMI. Per allele effects were obtained by linear regression with the allelic scores and FTO genotype. Effects were adjusted for age, and also for BMI in regressions involving genotype. *Sample size varies from 2,103 to 3,991 depending on completeness of data on confounding factors. ±Excluding dietary under-reporters. (DOCX) [file pmed.1001618.s007.docx]

| **Variable*** | **Body mass index (kg/m**^2^**)** | | | **Fat mass index (kg/m**^2^**)** | | | **Weighted allelic score with 32 SNPs** | | | **Weighted allelic score with 31 SNPs** | | | **FTO genotype** | | |
| --- | --- | --- | --- | --- | --- | --- | --- | --- | --- | --- | --- | --- | --- | --- | --- |
|  | **Coef** | **95%CI** | **P** | **Coef** | **95%CI** | **P** | **Coef** | **95%CI** | **P** | **Coef** | **95%CI** | **P** | **Coef** | **95%CI** | **P** |
| Daily dietary intake (kcal) | 4.31 | -7.10, 15.7 | 0.46 | -8.57 | -20.0, 2.88 | 0.14 | 3.14 | 0.15, 6.13 | 0.04 | -0.17 | -3.44, 3.10 | 0.92 | 34.6 | 18.2,51.0 | 3.4x10^-5^ |
| Daily dietary intake (kcal)^±^ | 139.8 | 126.9,152.9 | 1.2x10^-98^ | 121.6 | 108.2, 135.1 | 2.6x10^-70^ | 1.25 | -1.50, 4.00 | 0.37 | -0.56 | -3.55, 2.44 | 0.72 | 18.5 | 3.16,33.8 | 0.02 |
| Dairy (g/day) | -0.65 | -6.51, 5.21 | 0.83 | -5.06 | -11.0, 0.84 | 0.09 | -0.40 | -1.94, 1.13 | 0.61 | -1.17 | -2.85, 0.50 | 0.17 | 5.55 | -2.88, 14.0 | 0.20 |
| Fruit (g/day) | -2.74 | -5.13, -0.35 | 0.024 | -4.66 | -7.06, -2.26 | 1.4x10^-3^ | 0.51 | -0.12, 1.14 | 0.11 | 0.24 | -0.44, 0.93 | 0.49 | 3.40 | -0.05, 6.84 | 0.05 |
| Vegetables  (g/day) | -1.03 | -2.92, 0.85 | 0.28 | -1.45 | -3.33, 0.44 | 0.13 | 0.29 | -0.20, 0.78 | 0.25 | 0.29 | -0.24, 0.83 | 0.28 | 0.61 | -2.10, 3.32 | 0.66 |
| Cereals (g/day) | 3.05 | 0.29, 5.80 | 0.03 | 0.95 | -1.83, 3.72 | 0.50 | 0.18 | -0.55, 0.90 | 0.63 | -0.07 | -0.86, 0.71 | 0.85 | 2.48 | -1.48, 6.44 | 0.22 |
| Meat (g/day) | 4.05 | 2.03, 6.07 | 8.5x10^-5^ | 3.52 | 1.49, 5.55 | 6.9x10^-4^ | 0.19 | -0.34, 0.72 | 0.47 | 0.25 | -0.33, 0.82 | 0.40 | 1.48 | -2.92, 2.89 | 0.99 |
| Sweets (g/day) | -1.49 | -3.63, 0.66 | 0.17 | -2.11 | -4.26, 0.04 | 0.05 | 0.75 | 0.19, 1.31 | 0.01 | 0.31 | -0.38, 0.85 | 0.45 | 1.57 | 2.92, 9.08 | 1.4x10^-4^ |
| Sugary drinks (g/day) | 17.6 | 10.5, 26.2 | 9.6x10^-6^ | 18.4 | 10.5, 26.2 | 4.2x10^-6^ | -2.09 | -4.13, -0.05 | 0.05 | -0.69 | -2.92, 1.54 | 0.54 | -16.3 | -27.6, -5.1 | 4.2x10^-3^ |
| Protein  (g/day) | 0.95 | 0.48, 1.42 | 6.6x10^-5^ | 0.40 | -0.07, 0.87 | 0.09 | 0.12 | 0.00, 0.24 | 0.05 | 0.01 | -0.12, 0.15 | 0.84 | 1.16 | 0.49, 1.84 | 6.8x10^-4^ |
| Fat (g/day) | 0.18 | -0.42, 0.78 | 0.56 | -0.11 | -0.71, 0.49 | 0.72 | 0.15 | 0.00, 0.31 | 0.06 | -0.04 | -0.22, 0.13 | 0.62 | 1.99 | 1.13, 2.85 | 6.1x10^-6^ |
| Saturated fat (g/day) | -0.32 | -0.60, -0.03 | 0.03 | -0.41 | -0.70, -0.13 | 4.4x10^-3^ | 0.08 | 0.00, 0.15 | 0.04 | -0.01 | -0.09, 0.07 | 0.86 | 0.88 | 0.47, 1.29 | 2.2x10^-5^ |
| Monounsat-  urated fat (g/day) | 0.16 | -0.05, 0.38 | 0.14 | 0.08 | -0.14, 0.29 | 0.49 | 0.04 | -0.02, 0.09 | 0.18 | -0.02 | -0.08, 0.04 | 0.49 | 0.59 | 0.28, 0.90 | 1.7x10^-4^ |
| Polyunsat-  urated fat (g/day) | 0.24 | 0.09, 0.38 | 1.1x10^-3^ | 0.18 | 0.03, 0.32 | 0.02 | 0.02 | -0.02, 0.06 | 0.33 | -0.02 | -0.06, 0.02 | 0.41 | 0.34 | 0.14, 0.54 | 1.1x10^-3^ |
| Trans fatty acid (g/day) | -0.02 | -0.05, 0.01 | 0.29 | -0.03 | -0.06, 0.00 | 0.05 | 0.01 | 0.00, 0.01 | 0.16 | 0.00 | -0.01, 0.01 | 0.80 | 0.07 | 0.03, 0.12 | 1.9x10^-3^ |
| Dietary cholesterol (g/day) | 3.03 | 0.31, 5.74 | 0.03 | 1.80 | -0.93, 4.54 | 0.20 | 0.39 | -0.32, 1.10 | 0.29 | -0.21 | -0.99, 0.57 | 0.60 | 5.86 | 1.96, 9.77 | 3.3x10^-3^ |
| Carbohydrate (g/day) | -0.33 | -1.98, 1.32 | 0.69 | -2.49 | -4.15, -0.84 | 3.1x10^-3^ | 0.35 | -0.08, 0.79 | 0.11 | 0.05 | -0.42, 0.52 | 0.82 | 3.29 | 0.92, 5.66 | 0.01 |
| Total sugar  (g/day) | -1.54 | -2.74, -0.34 | 0.01 | -2.57 | -3.77, -1.36 | 3.0x10^-3^ | 0.09 | -0.22, 0.41 | 0.56 | -0.03 | -0.37, 0.31 | 0.86 | 1.25 | -0.47, 2.98 | 0.16 |
| Starch (g/day) | 1.29 | 0.37, 2.21 | 6.0x10^-3^ | 0.18 | -0.74, 1.10 | 0.70 | 0.25 | 0.01, 0.49 | 0.04 | 0.07 | -0.19, 0.34 | 0.58 | 2.01 | 0.69, 3.33 | 2.8x10^-3^ |
| Non-starch poly-  saccharide  (g/day) | -0.04 | -0.15, 0.07 | 0.52 | -0.17 | -0.29, -0.06 | 2.5x10^-3^ | 0.03 | 0.00, 0.06 | 0.03 | 0.01 | -0.02, 0.04 | 0.46 | 0.24 | 00.08, 0.40 | 4.0x10^-3^ |
