# Supplementary material for: Assessing Causality in the Association between Child Adiposity and Physical Activity Levels: A Mendelian Randomization Analysis
Source: PLoS Med. 2014 Mar 18;11(3):e1001618. doi: 10.1371/journal.pmed.1001618 (PMC3958348; doi:10.1371/journal.pmed.1001618)
Supplement: Table S6 — Associations between body mass index/fat mass index and activity levels as tested both by conventional epidemiological approaches and through the application of instrumental variable analysis using a 32-SNP weighted allelic score as an instrument at age 13 y. Regression results were adjusted for age. Coefficients are displayed as sex-specific z-scores for both measures of adiposity and activity levels. P(DWH) is the p-value of the Durbin form of the DWH test, which examines the difference between the estimates from linear regression and instrumental variable analysis. *Moderate-to-vigorous activity was log transformed for analysis. (DOCX) [file pmed.1001618.s008.docx]

| **Change per SD sex-specific body mass index** | | | | | | | | | | | | | |
| --- | --- | --- | --- | --- | --- | --- | --- | --- | --- | --- | --- | --- | --- |
| **BMI (n=2993)** | **Linear regression Coefficient** | **95% CI** | **P** | **Instrumental variable regression (weighted allelic score with 32 SNPs)** | | | **Coefficient** | **95% CI** | | **P** | | **P (DWH)** | |
|  |  |  |  | **F-statistic** | | **Partial R^2^** |  |  | |  | |  | |
| Total physical activity | -0.08 | -0.12, -0.04 | 1.4x10^-5^ | 97.64 | | 0.03 | -0.09 | -0.29, 0.11 | | 0.37 | | 0.91 | |
| Moderate-to-vigorous activity* | -0.09 | -0.13, -0.06 | 3.1x10^-7^ |  | | | -0.06 | -0.27, 0.13 | | 0.54 | | 0.77 | |
| Sedentary | 0.04 | 0.01, 0.08 | 0.02 |  | | | 0.07 | -0.12, 0.28 | | 0.47 | | 0.76 | |
| **Change per SD sex-specific fat mass index** | | | | | | | | | | | | | |
| **FMI (n=2965)** | **Linear regression Coefficient** | **95% CI** | **P** | **Instrumental variable regression (weighted allelic score with 32 SNPs)** | | | **Coefficient** | **95% CI** | | **P** | | **P (DWH)** | |
|  |  |  |  | **F-statistic** | **Partial R^2^** | |  | |  | |  | |  |
| Total physical activity | -0.14 | -0.17, -0.10 | 3.4x10^-14^ | 65.34 | 0.02 | | -0.11 | | -0.36, 0.13 | | 0.36 | | 0.84 |
| Moderate-to-vigorous activity* | -0.15 | -0.18, -0.11 | 6.4x10^-16^ |  | | | -0.08 | -0.32, 0.16 | | 0.52 | | 0.59 | |
| Sedentary | 0.08 | 0.05, 0.12 | 4.4x10^-6^ |  | | | 0.09 | -0.15, 0.33 | | 0.46 | | 0.95 | |
