# Supplementary material for: Assessing Causality in the Association between Child Adiposity and Physical Activity Levels: A Mendelian Randomization Analysis
Source: PLoS Med. 2014 Mar 18;11(3):e1001618. doi: 10.1371/journal.pmed.1001618 (PMC3958348; doi:10.1371/journal.pmed.1001618)
Supplement: Table S7 — Associations between genome-wide prediction scores and activity measures in independent subgroups. Regression results were adjusted for age. Per allele effects were obtained by linear regression for all of these continuous variables. Coefficients are based on z-scores for activity levels. *Moderate-to-vigorous activity was log transformed for analysis. (DOCX) [file pmed.1001618.s009.docx]

|  | **Per-allele effects** | | | | **Per-allele effects (adjusted for BMI)** | | | |
| --- | --- | --- | --- | --- | --- | --- | --- | --- |
| **Genotype** | **Outcome** | **Coefficient** | **SE** | **P** | **Outcome** | **Coefficient** | **SE** | **P** |
|  | **Subgroup 1 (n=2148)** | | | | | | | |
| Total physical activity prediction score | Total physical activity | 0.055 | 0.021 | 0.009 | Total physical activity | 0.057 | 0.021 | 0.007 |
| Moderate-to-vigorous activity* prediction score | Moderate-to-vigorous activity * | 0.051 | 0.021 | 0.018 | Moderate-to-vigorous activity * | 0.055 | 0.021 | 0.009 |
| Sedentary prediction score | Sedentary | 0.048 | 0.021 | 0.023 | Sedentary | 0.050 | 0.021 | 0.017 |
|  | **Subgroup 2(n=2148)** | | | | | | | |
| Total physical activity prediction score | Total physical activity | 0.036 | 0.022 | 0.078 | Total physical activity | 0.040 | 0.022 | 0.064 |
| Moderate-to-vigorous activity * prediction score | Moderate-to-vigorous activity * | 0.045 | 0.022 | 0.038 | Moderate-to-vigorous activity * | 0.041 | 0.021 | 0.055 |
| Sedentary prediction score | Sedentary | 0.047 | 0.022 | 0.033 | Sedentary | 0.048 | 0.022 | 0.030 |
