# Supplementary material for: Assessing Causality in the Association between Child Adiposity and Physical Activity Levels: A Mendelian Randomization Analysis
Source: PLoS Med. 2014 Mar 18;11(3):e1001618. doi: 10.1371/journal.pmed.1001618 (PMC3958348; doi:10.1371/journal.pmed.1001618)
Supplement: Table S8 — Correlations between genome-wide prediction scores and the BMI allelic score. Pearson product-moment correlation coefficients calculated. (DOCX) [file pmed.1001618.s010.docx]

|  | **Sample 1** | **Sample 2** |
| --- | --- | --- |
|  | BMI allele score | BMI allele score |
| Total physical activity prediction score | -0.007 | -0.018 |
| Moderate-to-vigorous activity  prediction score | -0.011 | 0.019 |
| Sedentary prediction score | 0.005 | 0.065 |
