# Supplementary material for: Assessing Causality in the Association between Child Adiposity and Physical Activity Levels: A Mendelian Randomization Analysis
Source: PLoS Med. 2014 Mar 18;11(3):e1001618. doi: 10.1371/journal.pmed.1001618 (PMC3958348; doi:10.1371/journal.pmed.1001618)
Supplement: Table S9 — Associations between activity levels and body mass index as tested both by conventional epidemiological approaches and through the application of instrumental variable analysis using genome-wide prediction scores for activity levels: meta-analysis for two sets of prediction scores. Regression results were adjusted for age. Coefficients are based on z-scores for activity and adiposity levels. P(DWH) is the p-value of the Durbin form of the DWH test, which examines the difference between the estimates from linear regression and instrumental variable analysis. *Moderate-to-vigorous activity was log transformed for analysis. $Physical activity prediction scores were generated in one subgroup and applied to individuals in a second independent subgroup for instrumental variable analysis. (DOCX) [file pmed.1001618.s011.docx]

| **Activity** | **Adiposity** | **N** | **Linear regression** | | | | | | **Instrumental variable regression (activity prediction scores**^§^**)** | | | | | | | | |
| --- | --- | --- | --- | --- | --- | --- | --- | --- | --- | --- | --- | --- | --- | --- | --- | --- | --- |
|  |  |  | **Coef** | **95% CI** | **P** | **Test of heterogeneity** | | | **F-statistic** | **Partial R^2^** | **Coef** | **95% CI** | **P** | **P (DWH)** | **Test of heterogeneity** | | |
|  |  |  |  |  |  | **Q** | | **P** |  |  |  |  |  |  | **Q** | | **P** |
| Total physical activity | Subgroup 1 BMI | 2148 | -0.14 | -0.19, -0.10 | 6.5x10^-11^ |  | | | 6.80 | 0.003 | 0.20 | -0.61, 1.01 | 0.62 | 0.38 |  | | |
|  | Subgroup 2 BMI | 2148 | -0.11 | -0.15, -0.07 | 2.6x10^-7^ |  | | | 3.10 | 0.001 | 0.41 | -0.81, 1.63 | 0.51 | 0.35 |  | | |
|  | Meta-analysis BMI | 4296 | -0.13 | -0.16 -0.10 | 1.7x10^-16^ | 1.37 | 0.24 | |  |  | 0.27 | -0.41, 0.94 | 0.44 |  | 0.08 | 0.78 | |
| Moderate-to-vigorous activity* | Subgroup 1 BMI | 2148 | -0.17 | -0.22, -0.13 | 6.8x10^-16^ |  |  | | 5.65 | 0.003 | 0.45 | -0.52, 1.42 | 0.36 | 0.14 |  | | |
|  | Subgroup 2 BMI | 2148 | -0.16 | -0.20, -0.12 | 7.8x10^-15^ |  | | | 4.31 | 0.002 | -0.52 | -1.50, 0.46 | 0.30 | 0.45 |  | | |
|  | Meta-analysis BMI | 4296 | -0.17 | -0.20, -0.14 | 4.0x10^-29^ | 0.13 | 0.72 | |  |  | -0.03 | -0.72, 0.66 | 0.93 |  | 1.90 | 0.17 | |
| Sedentary time | Subgroup 1 BMI | 2148 | 0.07 | 0.02, 0.12 | 0.11 |  | | | 5.20 | 0.002 | -0.69 | -1.80, 0.41 | 0.22 | 0.09 |  | | |
|  | Subgroup 2 BMI | 2148 | 0.04 | 0.00, 0.08 | 0.047 |  | | | 4.53 | 0.002 | -0.37 | -1.34, 0.60 | 0.45 | 0.36 |  | | |
|  | Meta-analysis BMI | 4296 | 0.05 | 0.02, 0.08 | 4.7x10^-4^ | 0.68 | 0.41 | |  |  | -0.51 | -1.24, 0.22 | 0.17 |  | 0.18 | 0.67 | |
