# Supplementary material for: Assessing Causality in the Association between Child Adiposity and Physical Activity Levels: A Mendelian Randomization Analysis
Source: PLoS Med. 2014 Mar 18;11(3):e1001618. doi: 10.1371/journal.pmed.1001618 (PMC3958348; doi:10.1371/journal.pmed.1001618)
Supplement: Table S10 — Associations between activity levels and fat mass index as tested both by conventional epidemiological approaches and through the application of instrumental variable analysis using genome-wide prediction scores for activity levels: meta-analysis for two sets of prediction scores. Regression results were adjusted for age. Coefficients are based on z-scores for activity and adiposity levels. P(DWH) is the p-value of the Durbin form of the DWH test, which examines the difference between the estimates from linear regression and instrumental variable analysis. *Moderate-to-vigorous activity was log transformed for analysis. $Physical activity prediction scores were generated in one subgroup and applied to individuals in a second independent subgroup for instrumental variable analysis. (DOCX) [file pmed.1001618.s012.docx]

| **Activity** | **Adiposity** | **N** | **Linear regression** | | | | | | **Instrumental variable regression (activity prediction scores**^§^**)** | | | | | | | | |
| --- | --- | --- | --- | --- | --- | --- | --- | --- | --- | --- | --- | --- | --- | --- | --- | --- | --- |
|  |  |  | **Coef** | **95% CI** | **P** | **Test of heterogeneity** | | | **F-statistic** | **Partial R^2^** | **Coef** | **95% CI** | **P** | **P (DWH)** | **Test of heterogeneity** | | |
|  |  |  |  |  |  | **Q** | | **P** |  |  |  |  |  |  | **Q** | | **P** |
| Total physical activity | Subgroup 1 FMI | 2116 | -0.20 | -0.24, -0.16 | 3.5x10^-20^ |  | | | 6.57 | 0.003 | 0.14 | -0.67, 0.96 | 0.73 | 0.38 |  | | |
|  | Subgroup 2 FMI | 2128 | -0.17 | -0.21, -0.13 | 2.6x10^-15^ |  | | | 3.03 | 0.001 | 0.25 | -0.94, 1.45 | 0.68 | 0.45 |  | | |
|  | Meta-analysis FMI | 4244 | -0.18 | -0.21, -0.15 | 1.3x10^-33^ | 1.21 | 0.27 | |  |  | 0.18 | -0.49, 0.85 | 0.60 |  | 0.02 | 0.88 | |
| Moderate-to-vigorous activity* | Subgroup 1 FMI | 2116 | -0.22 | -0.27**,** -0.18 | 2.0x10^-25^ |  |  | | 6.01 | 0.003 | 0.48 | -0.49**,** 1.45 | 0.33 | 0.08 |  | | |
|  | Subgroup 2 FMI | 2128 | -0.22 | -0.26**,** -0.18 | 1.4x10^-24^ |  | | | 4.00 | 0.002 | -0.75 | -1.84**,** 0.34 | 0.18 | 0.28 |  | | |
|  | Meta-analysis FMI | 4244 | -0.22 | -0.25**,** -0.19 | 2.5x10^-48^ | 0.04 | 0.83 | |  |  | -0.06 | -0.78**,** 0.66 | 0.87 |  | 2.73 | 0.11 | |
| Sedentary time | Subgroup 1 FMI | 2116 | 0.10 | 0.06, 0.15 | 6.8x10^-6^ |  | | | 5.07 | 0.002 | -0.72 | -1.86, 0.43 | 0.22 | 0.07 |  | | |
|  | Subgroup 2 FMI | 2128 | 0.08 | 0.04, 0.12 | 3.2x10^-4^ |  | | | 4.22 | 0.002 | -0.22 | -1.19, 0.75 | 0.66 | 0.53 |  | | |
|  | Meta-analysis FMI | 4244 | 0.09 | 0.06, 0.12 | 1.2x10^-8^ | 0.65 | 0.42 | |  |  | -0.43 | -1.17, 0.31 | 0.26 |  | 0.43 | 0.51 | |
