# Supplementary material for: Assessing Causality in the Association between Child Adiposity and Physical Activity Levels: A Mendelian Randomization Analysis
Source: PLoS Med. 2014 Mar 18;11(3):e1001618. doi: 10.1371/journal.pmed.1001618 (PMC3958348; doi:10.1371/journal.pmed.1001618)
Supplement: Table S11 — Associations between body mass index/fat mass index and activity levels as tested both by conventional epidemiological approaches and through the application of instrumental variable analysis using a 32-SNP weighted allelic score as an instrument: analysis stratified by sex. Regression results were adjusted for age. Coefficients are displayed as sex-specific z-scores for both measures of adiposity and activity levels. P(DWH) is the p-value of the Durbin form of the DWH test, which examines the difference between the estimates from linear regression and instrumental variable analysis. *Moderate-to-vigorous activity was log transformed for analysis. (DOCX) [file pmed.1001618.s013.docx]

| **Males** |  |  |  | | |  | | | | | |
| --- | --- | --- | --- | --- | --- | --- | --- | --- | --- | --- | --- |
| **Adiposity** | **Activity** | **N** | **Linear regression** | | | **Instrumental variable regression (weighted allelic score with 32 SNPs)** | | | | | |
|  |  |  | **Coefficient** | **95% CI** | **P** | **F-statistic** | **Partial R^2^** | **Coefficient** | **95% CI** | **P** | **P (DWH)** |
| Body mass index | Total physical activity | 2044 | -0.16 | -0.21, -0.12 | 9.3x10^-14^ | 63.1 | 0.03 | -0.17 | -0.42, 0.07 | 0.17 | 0.93 |
|  | Moderate-to-vigorous activity* |  | -0.23 | -0.27, -0.19 | 3.4x10^-26^ |  |  | -0.23 | -0.48, 0.01 | 0.06 | 0.97 |
|  | Sedentary time |  | 0.07 | 0.02, 0.11 | 0.003 |  |  | 0.14 | -0.11, 0.39 | 0.26 | 0.54 |
| Fat mass index | Total physical activity | 2022 | -0.22 | -0.26, 0.17 | 2.7x10^-23^ | 50.9 | 0.02 | -0.24 | -0.51, 0.03 | 0.09 | 0.88 |
|  | Moderate-to-vigorous activity* |  | -0.28 | -0.32, 0.24 | 1.7x10^-38^ |  |  | -0.30 | -0.56, -0.03 | 0.03 | 0.88 |
|  | Sedentary time |  | 0.10 | 0.06, 0.14 | 7.4x10^-6^ |  |  | 0.19 | -0.08, 0.45 | 0.16 | 0.48 |
| **Females** |  |  |  |  |  |  |  |  |  |  |  |
| **Adiposity** | **Activity** | **N** | **Linear regression** | | | **Instrumental variable regression (weighted allelic score with 32 SNPs)** | | | | | |
|  |  |  | **Coefficient** | **95% CI** | **P** | **F-statistic** | **Partial R^2^** | **Coefficient** | **95% CI** | **P** | **P (DWH)** |
| Body mass index | Total physical activity | 2252 | -0.09 | -0.13, -0.05 | 1.6x10^-5^ | 61.9 | 0.03 | -0.19 | -0.44, 0.06 | 0.14 | 0.44 |
|  | Moderate-to-vigorous activity* |  | -0.11 | -0.16, -0.07 | 4.4x10^-8^ |  |  | -0.13 | -0.38, 0.12 | 0.31 | 0.90 |
|  | Sedentary time |  | 0.04 | 0.00, 0.08 | 0.05 |  |  | 0.25 | 0.00, 0.50 | 0.06 | 0.10 |
| Fat mass index | Total physical activity | 2222 | -0.15 | -0.19, -0.11 | 5.4x10^-13^ | 57.4 | 0.03 | -0.17 | -0.43, 0.09 | 0.19 | 0.87 |
|  | Moderate-to-vigorous activity* |  | -0.16 | -0.20, -0.12 | 2.9x10^-15^ |  |  | -0.11 | -0.37, 0.14 | 0.38 | 0.70 |
|  | Sedentary time |  | 0.08 | 0.03, 0.12 | 3.7x10^-4^ |  |  | 0.24 | -0.03, 0.50 | 0.08 | 0.21 |
