# Supplementary material for: Assessing Causality in the Association between Child Adiposity and Physical Activity Levels: A Mendelian Randomization Analysis
Source: PLoS Med. 2014 Mar 18;11(3):e1001618. doi: 10.1371/journal.pmed.1001618 (PMC3958348; doi:10.1371/journal.pmed.1001618)
Supplement: Table S12 — Associations between activity levels and body mass index as tested both by conventional epidemiological approaches and through the application of instrumental variable analysis using genome-wide prediction scores for activity levels: analysis stratified by sex. Regression results were adjusted for age. Coefficients are based on z-scores for activity and adiposity levels. P(DWH) is the p-value of the Durbin form of the DWH test, which examines the difference between the estimates from linear regression and instrumental variable analysis. *Moderate-to-vigorous activity was log transformed for analysis. $Physical activity prediction scores were generated in one subgroup and applied to individuals in a second independent subgroup for instrumental variable analysis. (DOCX) [file pmed.1001618.s014.docx]

| **Males** | | | | | | | | | | | | | | | | |
| --- | --- | --- | --- | --- | --- | --- | --- | --- | --- | --- | --- | --- | --- | --- | --- | --- |
| **Activity** | **Adiposity** | **N** | **Linear regression** | | | | | **Instrumental variable regression (activity prediction scores**^§^**)** | | | | | | | | |
|  |  |  | **Coef** | **95% CI** | **P** | **Test of heterogeneity** | | **F-statistic** | **Partial R^2^** | **Coef** | **95% CI** | **P** | | **P (DWH)** | **Test of heterogeneity** | |
|  |  |  |  |  |  | **Q** | **P** |  |  |  |  |  |  |  | **Q** | **P** |
| Total physical activity | Subgroup 1 BMI | 1056 | -0.17 | -0.23, -0.11 | 3.9x10^-8^ |  | | 0.78 | 0.001 | 2.39 | -3.70, 8.47 | 0.44 | | 0.02 |  | |
|  | Subgroup 2 BMI | 988 | -0.16 | -0.22, -0.10 | 4.6x10^-7^ |  | | 2.30 | 0.002 | -0.27 | -1.54, 1.00 | 0.67 | | 0.86 |  | |
|  | Meta-analysis BMI | 2044 | -0.16 | -0.21, -0.12 | 9.2x10^-14^ | 0.09 | 0.77 |  |  | -0.16 | -1.40, 1.08 | 0.80 | |  | 0.70 | 0.40 |
| Moderate-to-vigorous activity | Subgroup 1 BMI | 1056 | -0.22 | -0.28, -0.16 | 5.9x10^-13^ |  | | 1.09 | 0.001 | 2.78 | -3.16, 8.73 | 0.36 | | 0.001 |  | |
|  | Subgroup 2 BMI | 988 | -0.23 | -0.29, -0.18 | 5.9x10^-15^ |  | | 4.13 | 0.004 | -0.60 | -1.57, 0.37 | 0.23 | | 0.43 |  | |
|  | Meta-analysis BMI | 2044 | -0.23 | -0.27, -0.19 | 2.4x10^-26^ | 0.07 | 0.80 |  |  | -0.51 | -1.47, 0.45 | 0.30 | |  | 1.21 | 0.99 |
| Sedentary time | Subgroup 1 BMI | 1056 | 0.07 | 0.01, 0.14 | 0.019 |  |  | 2.29 | 0.002 | -1.02 | -2.97, 0.92 | 0.30 | | 0.11 |  | |
|  | Subgroup 2 BMI | 988 | 0.06 | 0.00, 0.12 | 0.05 |  |  | 4.70 | 0.004 | 0.022 | -0.85, 0.90 | 0.96 | | 0.93 |  | |
|  | Meta-analysis BMI | 2044 | 0.07 | 0.02, 0.22 | 0.001 | 0.10 | 0.75 |  |  | -0.15 | -0.95, 0.65 | 0.71 | |  | 0.92 | 0.34 |
| **Females** | | | | | | | | | | | | | | | | |
| **Activity** | **Adiposity** | **N** | **Linear regression** | | | | | **Instrumental variable regression (activity prediction scores**^§^**)** | | | | | | | | |
|  |  |  | **Coef** | **95% CI** | **P** | **Test of heterogeneity** | | **F-statistic** | **Partial R^2^** | **Coef** | **95% CI** | | **P** | **P (DWH)** | **Test of heterogeneity** | |
|  |  |  |  |  |  | **Q** | **P** |  |  |  |  |  |  |  | **Q** | **P** |
| Total physical activity | Subgroup 1 BMI | 1092 | -0.12 | -0.18, -0.05 | 2.0x10^-4^ |  | | 7.74 | 0.007 | -0.49 | -1.27, 0.28 | | 0.21 | 0.31 |  | |
|  | Subgroup 2 BMI | 1160 | -0.07 | -0.13, -0.01 | 0.01 |  | | 1.05 | 0.001 | 1.29 | -1.89, 4.47 | | 0.43 | 0.12 |  | |
|  | Meta-analysis BMI | 2252 | -0.09 | -0.13, -0.05 | 1.5x10^-5^ | 1.19 | 0.28 |  |  | -0.39 | -1.16, 0.36 | | 0.31 |  | 1.14 | 0.29 |
| Moderate-to-vigorous activity | Subgroup 1 BMI | 1092 | -0.13 | -0.19, -0.07 | 2.1x10^-5^ |  | | 13.93 | 0.01 | -0.33 | -0.87, 0.20 | | 0.22 | 0.44 |  | |
|  | Subgroup 2 BMI | 1160 | -0.10 | -0.16, -0.04 | 4.7x10^-4^ |  | | 0.74 | 0.001 | 0.18 | -2.26, 2.30 | | 0.99 | 0.92 |  | |
|  | Meta-analysis BMI | 2252 | -0.12 | -0.16, -0.07 | 4.3x10^-8^ | 0.37 | 0.54 |  |  | -0.31 | -0.83, 0.21 | | 0.24 |  | 0.09 | 0.77 |
| Sedentary time | Subgroup 1 BMI | 1092 | 0.06 | 0.00, 0.12 | 0.06 |  | | 3.00 | 0.002 | -0.39 | -1.67, 0.88 | | 0.55 | 0.45 |  | |
|  | Subgroup 2 BMI | 1160 | 0.03 | -0.03, 0.08 | 0.35 |  | | 0.80 | 0.001 | -1.24 | -4.72, 2.24 | | 0.49 | 0.24 |  | |
|  | Meta-analysis BMI | 2252 | 0.04 | 0.00, 0.08 | 0.05 | 0.61 | 0.44 |  |  | -0.50 | -1.70, 0.71 | | 0.42 |  | 0.20 | 0.66 |
