# Supplementary material for: Assessing Causality in the Association between Child Adiposity and Physical Activity Levels: A Mendelian Randomization Analysis
Source: PLoS Med. 2014 Mar 18;11(3):e1001618. doi: 10.1371/journal.pmed.1001618 (PMC3958348; doi:10.1371/journal.pmed.1001618)
Supplement: Table S13 — Associations between activity levels and fat mass index as tested both by conventional epidemiological approaches and through the application of instrumental variable analysis using genome-wide prediction scores for activity levels: meta-analysis for two sets of prediction scores stratified by sex. Regression results were adjusted for age. P(DWH) is the p-value of the Durbin form of the DWH test, which examines the difference between the estimates from linear regression and instrumental variable analysis. *Moderate-to-vigorous activity was log transformed for analysis. $Physical activity prediction scores were generated in one subgroup and applied to individuals in a second independent subgroup for instrumental variable analysis. (DOCX) [file pmed.1001618.s015.docx]

| **Males** | | | | | | | | | | | | | | | | | | | | | |
| --- | --- | --- | --- | --- | --- | --- | --- | --- | --- | --- | --- | --- | --- | --- | --- | --- | --- | --- | --- | --- | --- |
| **Activity** | **Adiposity** | **N** | **Linear regression** | | | | | | | | | **Instrumental variable regression (activity prediction scores**^§^**)** | | | | | | | | | |
|  |  |  | **Coef** | **95% CI** | | **P** | | **Test of heterogeneity** | | | | **F-statistic** | | **Partial R^2^** | | **Coef** | **95% CI** | **P** | **P (DWH)** | **Test of heterogeneity** | |
|  |  |  |  |  |  |  |  | **Q** | | **P** | |  |  |  |  |  |  |  |  | **Q** | **P** |
| Total physical activity | Subgroup 1 FMI | 1042 | -0.23 | -0.29, -0.17 | | 4.3x10^-14^ | |  | | | | 0.58 | | 0.001 | | 2.35 | -4.76, 9.47 | 0.52 | 0.05 |  | |
|  | Subgroup 2 FMI | 980 | -0.20 | -0.26, -0.14 | | 8.7x10^-11^ | |  | | | | 2.59 | | 0.003 | | -0.43 | -1.65, 0.78 | 0.48 | 0.70 |  | |
|  | Meta-analysis FMI | 2022 | -0.22 | -0.26, -0.17 | | 3.1x10^-23^ | | 0.54 | | 0.46 | |  | |  | | -0.36 | -1.55, 0.84 | 0.56 |  | 0.57 | 0.45 |
| Moderate-to-vigorous activity | Subgroup 1 FMI | 1042 | -0.28 | -0.34, -0.22 | | 5.0x10^-20^ | |  | | | | 1.09 | | 0.001 | | 2.30 | -2.88, 7.47 | 0.38 | 0.01 |  | |
|  | Subgroup 2 FMI | 980 | -0.27 | -0.33, -0.22 | | 3.8x10^-20^ | |  | | | | 4.45 | | 0.005 | | -0.62 | -1.54, 0.31 | 0.19 | 0.44 |  | |
|  | Meta-analysis FMI | 2022 | -0.28 | -0.32, -0.24 | | 1.6x10^-38^ | | 0.03 | | 0.87 | |  | |  | | -0.53 | -1.43, 0.38 | 0.26 |  | 1.18 | 0.65 |
| Sedentary time | Subgroup 1 FMI | 1042 | 0.11 | 0.05, 0.17 | | 7.6x10^-4^ | |  | | | | 2.36 | | 0.002 | | -0.76 | -2.48, 0.96 | 0.39 | 0.20 |  | |
|  | Subgroup 2 FMI | 980 | 0.09 | 0.03, 0.15 | | 0.003 | |  | | | | 4.99 | | 0.005 | | 0.16 | -0.68, 1.01 | 0.71 | 0.88 |  | |
|  | Meta-analysis FMI | 2022 | 0.10 | 0.06, 0.14 | | 6.5x10^-6^ | | 0.11 | | 0.74 | |  | |  | | -0.02 | -0.78, 0.74 | 0.96 |  | 0.88 | 0.35 |
| **Females** | | | | | | | | | | | | | | | | | | | | | |
| **Activity** | **Adiposity** | **N** | **Linear regression** | | | | | | | | | | **Instrumental variable regression (activity prediction scores**^§^**)** | | | | | | | | |
|  |  |  | **Coef** | | **95% CI** | | **P** | | **Test of heterogeneity** | | | | **F-statistic** | | **Partial R^2^** | **Coef** | **95% CI** | **P** | **P (DWH)** | **Test of heterogeneity** | |
|  |  |  |  | |  | |  | | **Q** | | **P** | |  | |  |  |  |  |  | **Q** | **P** |
| Total physical activity | Subgroup 1 FMI | 1074 | -0.17 | | -0.23, -0.11 | | 6.9x10^-8^ | |  | | | | 8.04 | | 0.008 | -0.45 | -1.18, 0.28 | 0.23 | 0.43 |  | |
|  | Subgroup 2 FMI | 1148 | -0.14 | | -0.20, -0.08 | | 1.2x10^-6^ | |  | | | | 0.83 | | 0.001 | 1.33 | -2.47, 5.14 | 0.49 | 0.17 |  | |
|  | Meta-analysis FMI | 2222 | -0.15 | | -0.20, -0.11 | | 4.7x10^-13^ | | 0.38 | | 0.54 | |  | |  | -0.38 | -1.10, 0.33 | 0.29 |  | 0.81 | 0.37 |
| Moderate-to-vigorous activity | Subgroup 1 FMI | 1074 | -0.17 | | -0.23, -0.11 | | 2.3x10^-8^ | |  | | | | 14.66 | | 0.14 | -0.29 | -0.80, 0.22 | 0.26 | 0.63 |  | |
|  | Subgroup 2 FMI | 1148 | -0.17 | | -0.23, -0.11 | | 2.4x10^-8^ | |  | | | | 0.57 | | 0.001 | -0.15 | -2.77, 2.47 | 0.91 | 0.99 |  | |
|  | Meta-analysis FMI | 2222 | -0.17 | | -0.21, -0.13 | | 2.9x10^-15^ | | 0.00 | | 0.99 | |  | |  | -0.28 | -0.78, 0.21 | 0.26 |  | 0.01 | 0.92 |
| Sedentary time | Subgroup 1 FMI | 1074 | 0.09 | | 0.03, 0.15 | | 0.003 | |  | | | | 2.81 | | 0.003 | -0.66 | -2.15, 0.83 | 0.38 | 0.22 |  | |
|  | Subgroup 2 FMI | 1148 | 0.06 | | 0.01, 0.12 | | 0.03 | |  | | | | 0.54 | | 0.001 | -1.24 | -5.57, 3.09 | 0.57 | 0.32 |  | |
|  | Meta-analysis FMI | 2222 | 0.08 | | 0.04, 0.12 | | 3.2x10^-4^ | | 0.53 | | 0.47 | |  | |  | -0.73 | -2.1, 0.69 | 0.31 |  | 0.06 | 0.80 |
